# A versatile delivery vehicle for cellular oxygen and fuels or metabolic sensor? A review and perspective on the functions of myoglobin

**DOI:** 10.1152/physrev.00031.2023

**Published:** 2024-05-02

**Authors:** Kiran Kumar Adepu, Andriy Anishkin, Sean H. Adams, Sree V. Chintapalli

**Affiliations:** ^1^Arkansas Children’s Nutrition Center and Department of Pediatrics, University of Arkansas for Medical Sciences, Little Rock, Arkansas, United States; ^2^Department of Biology, University of Maryland, College Park, Maryland, United States; ^3^Department of Surgery, School of Medicine, University of California Davis, Sacramento, California, United States; ^4^Center for Alimentary and Metabolic Science, School of Medicine, University of California Davis, Sacramento, California, United States

**Keywords:** fatty acid binding protein, lipid and metabolite trafficking, metabolite binding protein, nitric oxide regulation, oxygen sensing

## Abstract

A canonical view of the primary physiological function of myoglobin (Mb) is that it is an oxygen (O_2_) storage protein supporting mitochondrial oxidative phosphorylation, especially as the tissue O_2_ partial pressure (Po_2_) drops and Mb off-loads O_2_. Besides O_2_ storage/transport, recent findings support functions for Mb in lipid trafficking and sequestration, interacting with cellular glycolytic metabolites such as lactate (LAC) and pyruvate (PYR), and “ectopic” expression in some types of cancer cells and in brown adipose tissue (BAT). Data from Mb knockout (Mb^−/−^) mice and biochemical models suggest additional metabolic roles for Mb, especially regulation of nitric oxide (NO) pools, modulation of BAT bioenergetics, thermogenesis, and lipid storage phenotypes. From these and other findings in the literature over many decades, Mb’s function is not confined to delivering O_2_ in support of oxidative phosphorylation but may serve as an O_2_ sensor that modulates intracellular Po_2_- and NO-responsive molecular signaling pathways. This paradigm reflects a fundamental change in how oxidative metabolism and cell regulation are viewed in Mb-expressing cells such as skeletal muscle, heart, brown adipocytes, and select cancer cells. Here, we review historic and emerging views related to the physiological roles for Mb and present working models illustrating the possible importance of interactions between Mb, gases, and small-molecule metabolites in regulation of cell signaling and bioenergetics.

## Graphical abstract

**Figure:**
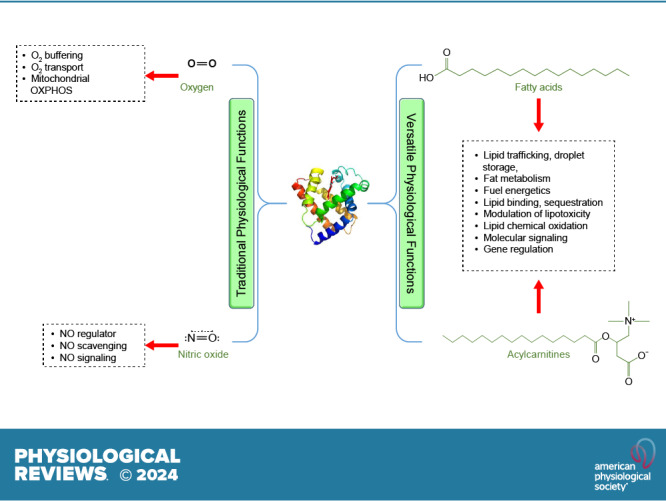

CLINICAL HIGHLIGHTS

- Myoglobin (Mb) is a globin protein family member primarily expressed in cardiac and skeletal muscle tissues. It has a well-known oxygen (O_2_) carrying and storage function, traditionally believed to support mitochondrial oxidative metabolism during increased O_2_ demand.
- Myoglobin knockout (Mb^−/−^) mice sustain cardiac muscle oxidative metabolism under normoxic conditions and develop compensatory adaptations such as increased capillarity, presumably to reduce the path length for O_2_ between the blood and the mitochondria. Although inconclusive and highly variable, in Mb^−/−^ mice there are some reports of modest effects on muscle performance, mitochondrial content, and fiber type transition.
- Biochemical and tissue-level studies support the idea that Mb is an important regulator of cellular nitric oxide (NO) pools, through O_2_-dependent mechanisms in which Mb is a NO scavenger during normoxia but functions as a NO producer (and thus possibly dampening the electron transport chain) as Po_2_ drops.
- Emerging models of Mb physiological function indicate that Mb serves as a “metabolic sensor” that regulates downstream molecular signaling pathways and gene expression, ultimately impacting metabolic status of the cell, i.e., leading to alterations in metabolic efficiency, thermogenic activation, and lipid trafficking in brown adipose tissue (BAT).
- Recent findings provide additional evidence to support a role for Mb in lipid and nonlipid metabolite binding, trafficking, and sequestration.

## 1. INTRODUCTION

**FIGURE 1.**
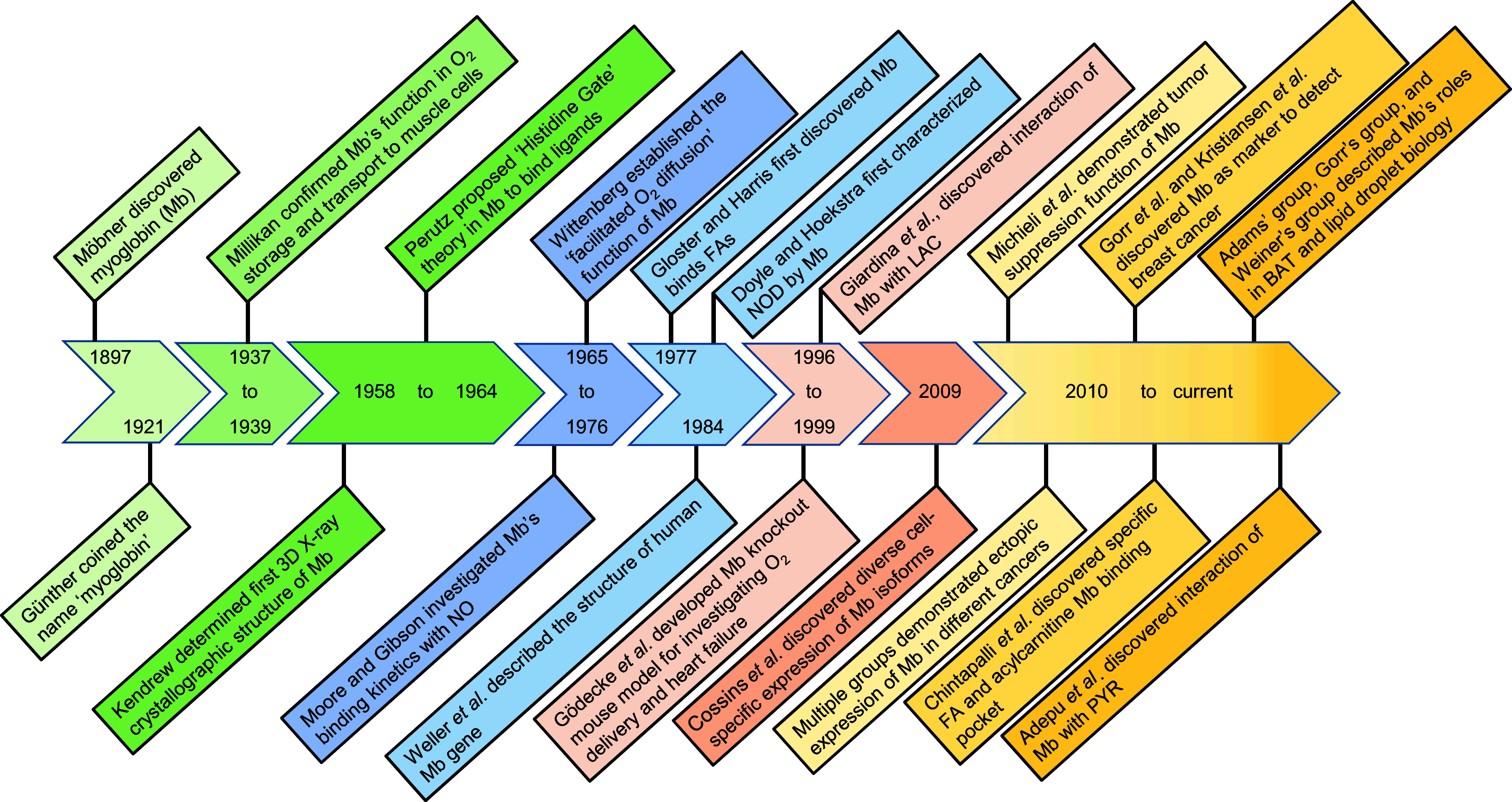
Historical timeline representation of some significant findings in myoglobin (Mb) research. The figure highlights some of the key studies and achievements of Mb research over 110+ years, including structural and biochemical characterizations, interactions with gas molecules, phenotypes in knockout mice, potential roles in tumor and brown adipocyte biology, and metabolite binding. 3D, 3-dimensional; BAT, brown adipose tissue; FA, fatty acid; LAC, lactate; NO, nitric oxide; NOD, NO dioxygenase; O_2_, oxygen; PYR, pyruvate. References cited in the figure are Mörner (5), Günther (2), Millikan (3), Kendrew et al. (4), Perutz et al. (6), Wittenburg (7), Moore and Gibson (8), Gloster and Harris (9), Doyle and Hoekstra (10), Weller et al. (11), Giardina et al. (12), Gödecke et al. (13), Cossins et al. (14), Michieli and colleagues. (15), Kristiansen et al. (16), Gorr et al. (17), Chintapalli et al. (18), Blackburn et al. (19), Aboouf et al. (20), Christen et al. (21), and Adepu et al. (22).

Myoglobin (Mb) was first discovered as a muscle pigment by spectroscopy, and a suggested name, myochrome, was proposed in 1897 to differentiate the protein from hemoglobin (1). Günther (2) confirmed the results in 1921 and renamed the protein as myoglobin. The O_2_ binding and storage function of Mb was discovered at the turn of the twentieth century (3) and has over six decades of detailed structural investigation (4) (FIGURE 1). In vertebrates, Mb is primarily expressed in cardiac and skeletal muscle cells (23–25), with highest levels in type I “oxidative” myofibers and less expression in type II “glycolytic” myofibers (26–28); that said, the latter reportedly contain significant Mb levels in human skeletal muscle fibers (29). Mb concentration in muscle varies with species, ranging from ∼150–270 µM in murine (3–8 mg/g) and human (5–10 mg/g) hearts (28, 30, 31) to ∼360–800 µM in horse quadriceps (10–20 mg/g) and human muscles (7–14 mg/g) (26, 28, 31–33). The concentration of muscle Mb in deep diving mammals (40–80 mg/g) (34, 35) and diving birds (60–70 mg/g) (36, 37) is far higher than that in terrestrial mammals (38–41). For instance, and with the caveat that measurements were from different muscle groups, Mb concentrations are very high in sperm whales (∼70–80 mg/g) (35), northern elephant seals (21–59 mg/g), and bottlenose dolphins (10–40 mg/g) compared to New Zealand white rabbits (10–20 mg/g) and mice (3–8 mg/g) (34, 42, 43) (assumes in some cases 1 mL/g wet tissue and 70% tissue hydration). Detectable but low levels of Mb were also identified in human smooth muscle cells (44).

Despite being the first protein to have a determination of crystallographic three-dimensional structure (4) and decades of biochemistry research, the full suite of physiological functions of Mb in cellular metabolism continues to be elaborated. Beyond a role in O_2_ storage, Mb has been implicated in other roles such as nitric oxide (NO) scavenging and NO generation (23, 45–51), long-chain fatty acid (LCFA) and long-chain acylcarnitine (LCAC) binding (9, 18, 52–58), and Mb-induced lipid peroxidation (59–66), to name a few. A large number of whole body metabolic studies in Mb knockout (Mb^−/−^) mice indicate that oxidative phosphorylation can be sustained despite loss of Mb (13, 67–71). In the present review, these and other relevant findings are described, highlighting emerging and novel perspectives for Mb functions. To place these perspectives into context, and to consider molecular actions and regulation of Mb, we first provide an overview of structural and biochemical characteristics of the protein.

## 2. MYOGLOBIN: STRUCTURAL BIOCHEMISTRY

### 2.1. Mb Is a Globin Family Protein

Mb is a member of the globin protein superfamily along with hemoglobin (Hb), neuroglobin (Ngb), cytoglobin (Cygb), and others. Most globin proteins are composed of ∼140–160 amino acids (molecular mass 15–18 kDa) and possess a globular fold containing a heme moiety as the prosthetic group. Blood Hb, with its well-known role as an O_2_ carrier and easy accessibility, overshadowed Mb as a research subject until Theorell’s successful isolation and crystallization of Mb in 1932 (3, 72). Theorell (73–76) conducted a comprehensive study on crystalline horse muscle Mb and its derivatives, covering a wide spectral range. Both Theorell (72–76) and Hill (77, 78) independently investigated gas-binding properties by different methods and found hyperbolic curves, high O_2_ affinity, and minimal pH effect on affinity, indicating agreement in their findings. Theorell (74) also studied the impact of temperature, revealing a lower heat of reaction compared to blood Hb. Unlike mammalian globins, an additional domain with flavin adenine nucleotide (FAD) and nicotinamide adenine dinucleotide phosphate [NAD(P)H] binding sites is found in a few classes of microbial globins (e.g., flavohemoglobins) (79, 80). Mb is also present in many invertebrates, with a greater structural and functional variability, and lower concentrations, than in vertebrates (81–84). Among the invertebrate globins, the functional significance of Mb in gastropods has been extensively studied (85–89).

**FIGURE 2.**
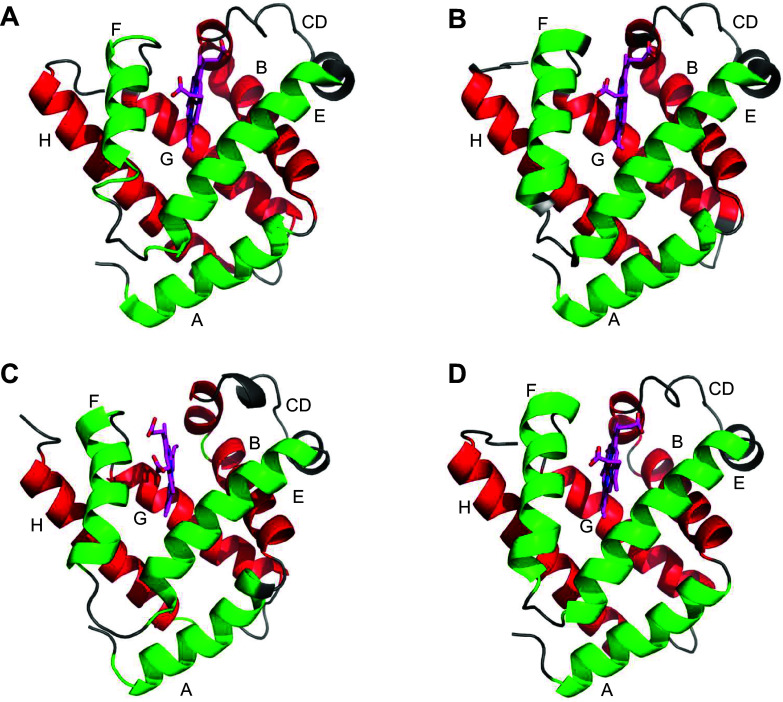
Myoglobin (Mb) structure displaying classical globin fold (a 3-on-3 α-helical sandwich). Snapshots of Mb structures from different species are shown. *A*: crystal structure of sperm whale Mb (PDB id: 1mbo). *B*: crystal structure of equine heart Mb (PDB id: 1wla). *C*: modeled structure of mouse Mb. *D*: modeled structure of human Mb. Protein structures of mouse Mb and human Mb were generated with ITASSER software (https://zhanggroup.org/I-TASSER/). The protein backbone is represented as ribbon shape (colored green and red), and the heme center is represented as sticks (colored magenta).

Mb is a compact helical protein (molecular mass ∼17 kDa) possessing eight α-helices (A–H) made up of 152 amino acids (4). Even distantly related Mb proteins share a highly conserved, compact, and rigid structural pattern, regarded as the “classical” globin fold (a 3-on-3 α-helical sandwich) (90), as illustrated in FIGURE 2. The helical pairs B/E and G/H of Mb are each arranged in an antiparallel manner that are assembled in a sort of α-helical bundle surrounding the heme group, and the two last helices (G/H) of the globin chain form a helix-turn-helix motif. In a majority of proteins antiparallel helices exhibit an ∼20° angle, whereas in globin family proteins (GFPs) the angle is ∼50°, as the helices pack against each other (91). This might be important in ligand-binding proteins such as Mb, where the side chain fluctuations facilitate ligand penetration from the surrounding solvent through the protein matrix to the active iron center and these fluctuations, in turn, affect functionally significant collective motions (92). Based on the coordination of the iron (Fe) atom by either one or two of the histidine (His) side chains, GFPs have been divided into two major classes: “penta-coordinate” and “hexa-coordinate” structures. In penta-coordinate structures like Mb and Hb, only the proximal His is attached to the heme Fe center and coordination of the distal His is reversible, allowing for binding of exogenous small-molecule ligands. In addition to the well‐investigated Mb and Hb proteins, genome sequence analyses in vertebrates have led to the identification of six other globin types: androglobin, Cygb, Ngb, globin E, globin X, and globin Y (93). Cygb and Ngb belong to the hexa-coordinate structural class, where both the distal and proximal His(s) are coordinated to the heme prosthetic group. All known GFPs are capable of reversible O_2_ binding. Besides O_2_ binding, hexa-coordinate GFPs like Cygb and Ngb have shed light on an alternate globin mechanism for modulating ligand binding. Cygb acts like a NO dioxygenase (NOD), and peroxidase enzyme (94) and Ngb act as allosteric proteins (95).

### 2.2. Role of the Mb Heme Center in Gas Binding and Kinetics

**FIGURE 3.**
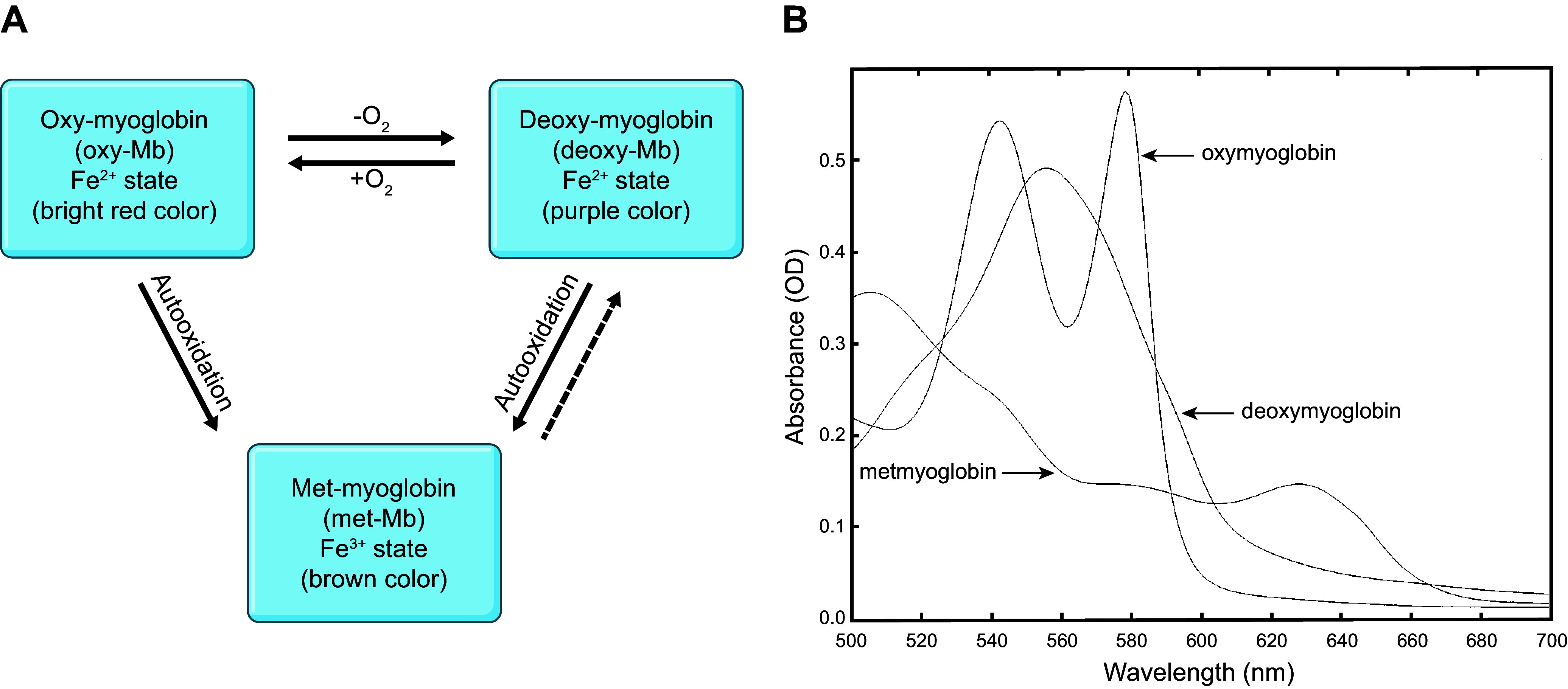
Graphical representation of different states of myoglobin (Mb) in a cell. *A*: the existence of major forms of Mb (oxy-Mb, deoxy-Mb, and met-Mb) upon interaction with gaseous molecules and their iron state. *B*: UV-Vis spectra displaying unique peak profiles of Mb forms. OD, optical density. Image reproduced from Ref. 97, with permission from the American Physiological Society.

Like other globins, Mb contains a heme group with a porphyrin ring-bound Fe atom at the center. X-ray diffraction studies of the three-dimensional crystal structure of Mb revealed that the polypeptide chain is folded and cradles the heme prosthetic group (4). The Fe^2+^ ion in Mb has six coordination sites. Four of these sites are equatorial and are occupied by four nitrogen atoms from the porphyrin ring. The remaining two sites are axial: one is occupied by a His residue (His^93^); the other axial site is available for binding to a variety of gas ligands, such as O_2_ in oxygenated Mb (oxy-Mb), nitric oxide (NO) (10, 46, 47), or carbon monoxide (CO) (96). However, in deoxygenated Mb (deoxy-Mb), the sixth coordination site is vacant. The various states of Mb are shown in FIGURE 3. The positioning of the heme group between the two key His residues, distal His^64^ and proximal His^93^, is crucial (98). For illustration, His^93^ binds the Fe^2+^ (reduced form) of the heme, and this stabilizes the heme group. His^64^ helps prevent oxidation (Fe^2+^ conversion to Fe^3+^) of the heme center and also decreases the binding affinity of CO to the heme (96). In addition to CO binding to Hb (99) and cytochrome *c* oxidase (CcOX) (100), high exposure to CO promotes avid binding to Mb and concomitantly lowers affinity for O_2_ binding to the Fe^2+^ heme center (96).

In the presence of excess CO, its binding to Mb may impair cardiac output (101) and promote hypotension (102) resulting in cerebral ischemia and finally acute CO poisoning (103). His^64^ plays a key role in the discrimination between O_2_ and CO binding, where His^64^ favors O_2_ binding by forming a robust hydrogen bond with O_2_, which carries a partial negative charge in the Fe(II) O_2_ complex. Conversely, the Fe(II) CO complex is electrically neutral, resulting in weak interaction with His^64^. Moreover, in deoxy-Mb, His^64^ impedes the binding of all ligands by hydrogen bonding to a distal pocket H_2_O molecule, necessitating its displacement before ligands can bind. This process reduces ligand affinities by ∼5–10 times. Specifically, the affinity for O_2_ is enhanced by 1,000-fold because of H-bonding to His^64^, resulting in a net increase of 100- to 200-fold in O_2_ affinity. In contrast, H-bonding to His^64^ only marginally stabilizes bound CO by two- to threefold, insufficient to counterbalance the requirement to displace the distal pocket water molecule in deoxy-Mb. Consequently, CO affinity decreases by fivefold compared to O_2_ (33, 104–106).

**FIGURE 4.**
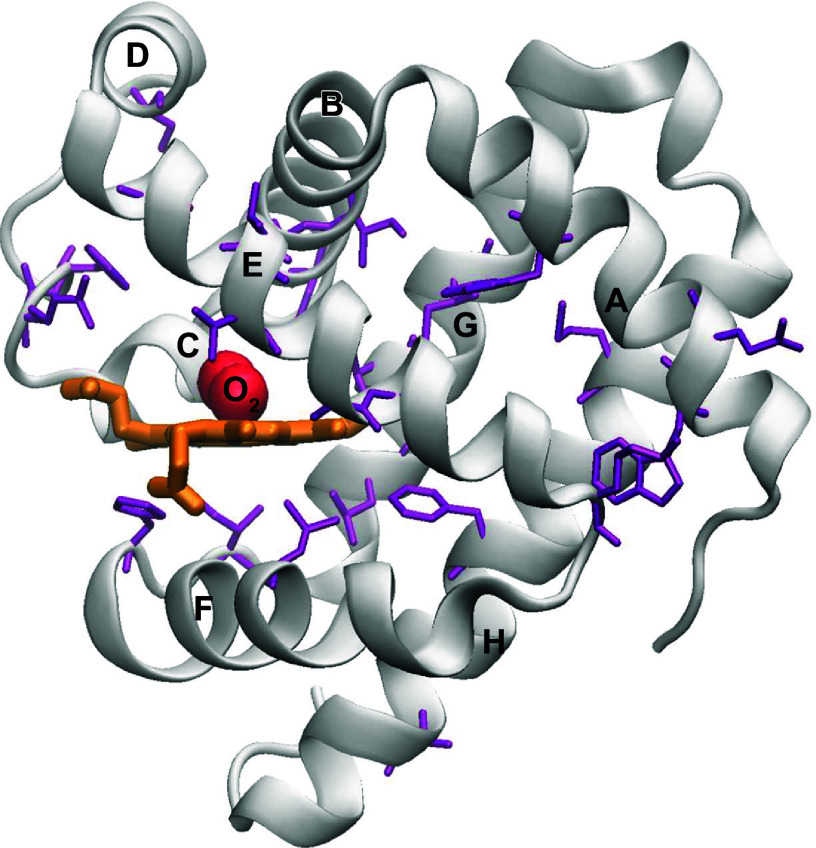
Cartoon representation of the structure of sperm whale metmyoglobin (met-Mb). The backbone is drawn as silver ribbons, and the amino acid side chains drawn as purple sticks mark the 27 native residues that were mutated; heme is represented as Licorice (orange), and O_2_ is displayed in VDW (red) colors. The helices are labeled in capital letters (A–H). Image adapted from Ref. 111 from the *Journal of Biological Chemistry* per CC-BY 4.0 Open Access.

In Mb, His-E7 acts as a gate known as the “His gate” with an open or closed conformation, controlling access of gas ligands to the active site (107–109). At neutral pH, molecular dynamics (MD) simulations suggest that Mb undergoes a transient conformational change from a closed to an open state, which involves a small outward rotation of the His^64^ side chain that enables O_2_ entry into the heme pocket and facilitates efficient O_2_ uptake by Mb (110). This idea is supported by dramatic increases in the rates of ligand binding at low pH, when His^64^ is protonated and rotates out into solvent, creating an empty channel into the distal pocket (108). Similar large increases in rates of entry and escape of gaseous ligands are observed when His^64^ (E7) and Phe^46^ (CD4) are replaced with small apolar amino acids or Gly, and vice versa. Substantially, large decreases in rates are observed with Trp and Phe mutations that block the E7 channel at these positions (FIGURE 4) (33, 47, 111–113). In another study, a significant decrease in O_2_ affinity and a ∼100 times increase in O_2_ dissociation rate constant was seen when His-E7 of Mb was mutated to Gly (98). This specific mutation resulted in a fivefold increase in CO affinity due to an increase in association rate constant that favors CO over O_2_.

## 3. ROLE OF Mb FOR SUPPLYING OXYGEN TO SUPPORT OXIDATIVE METABOLISM: A CLASSIC VIEW OF Mb FUNCTION

### 3.1. O_2_ Management and Transport

Historically, Mb has primarily been considered in terms of myocyte or cardiomyocyte O_2_ storage, Po_2_-dependent O_2_ buffering, and facilitated O_2_ diffusion to support oxidative metabolism (114). In support of this view, during rigorous strenuous exercise or increased O_2_ demand, Po_2_ drops and Mb becomes increasingly deoxygenated (115–119). Mb is thought to transfer O_2_ to the mitochondria of heart and red muscle cells (31, 120–124). That said, when freshly extracted Mb preparations are tested in vitro under physiological conditions, it is difficult to determine accurate Mb oxygen affinity (P_50_). For instance, in Mb preparations from bovine heart muscle, pig, or sperm whale, there is rapid autooxidation of Mb, where the Fe^2+^ in the heme center reacts with molecular O_2_ to form Fe^3+^ ion and superoxide radicals (O_2_^•−^) to yield the metmyoglobin form (met-Mb, oxidized form of Mb where heme iron is in the Fe^3+^ state) (125, 126); the latter is incapable of binding O_2_ (127). Autooxidation occurs through a combination of two mechanisms: O_2_^•−^ dissociation and a bimolecular reaction with O_2_ (126). The rate of autooxidation of Mb is dependent on O_2_ concentration. At high O_2_ concentrations, the autooxidation rate is primarily influenced by the dissociation of the neutral O_2_^•−^ radical from oxy-Mb. This process is accelerated by decreasing pH. Conversely, at low O_2_ concentrations, autooxidation occurs through a bimolecular reaction between molecular O_2_ and deoxy-Mb containing a weakly coordinated H_2_O molecule. The neutral side chain of the distal His inhibits autooxidation by forming H-bonds to bound O_2_, preventing both O_2_^•−^ dissociation and the oxidative bimolecular reaction with deoxy-Mb.

Additionally, the rate of Mb autooxidation to met-Mb is temperature and pH dependent, where hemin dissociation (loss of heme group) leads to apoglobin formation and is often observed resulting in loss of Mb’s native structure and function (126, 128). For example, in *Bos taurus* skeletal muscle Mb preparations, compared with extraction buffer at pH 7.4 and 37°C, buffer at pH 8.26 and 5°C effectively removed Hb contamination and also decreased Mb autooxidation (127). Similarly, lower autooxidation rates of bovine, ovine, porcine, and cervine oxy-Mb were recorded as temperature was reduced from 40°C to 20°C during Mb extraction and also when buffer pH was increased from pH 5.5 to 6.5 (129). The autooxidation reaction can lead to the formation of reactive oxygen species (ROS) and subsequent oxidative damage of Mb. His^64^, Val^68^, and Leu^29^ amino acids are pivotal in modulating the autooxidation rate by affecting the polarity and size of the distal pocket, thereby impacting the accessibility of the Fe^2+^ atom to solvent H_2_O molecules. Moreover, the evolutionary pressure to preserve Leu29 [vs. Tyr (130) or Phe (131)] at the B10 position in Mb likely signifies a balance between minimizing the rate of autooxidation and ensuring a sufficiently high O_2_ dissociation rate constant to facilitate swift O_2_ release during respiration (131). These findings provide valuable insights into the factors influencing the autooxidation process of Mb.

### 3.2. Mb-Mitochondria Interactions and the Potential Role of Mb in Supporting Mitochondrial Respiration

Mitochondrial respiration in working muscle or heart requires constant O_2_ supply for oxidative phosphorylation in response to increased energy demands. During the process of transport and delivery of O_2_ in cardiac muscle cells, a concentration equilibrium exists between the oxy-Mb and deoxy-Mb near the mitochondrial membrane with concurrent ATP generation through electron transport chain, favoring an O_2_ diffusion flux into the mitochondria (132). Immunohistochemical and electron microscopy analyses show the colocalization of Mb with mitochondria and close interaction with CcOX subunit IV (CcOX-IV) (133). Mb was shown to interact with mitochondrial membrane (134), and deoxy-Mb-derived NO inhibits CcOX-IV (135, 136) through S-nitrosation of a cysteine by binding of NO^•^ to the CuB/haem-a3 site in mitochondrial complex I (137). However, there is no direct molecular evidence of Mb-mediated S-nitrosation on mitochondrial complex IV. Furthermore, during the O_2_ exchange from Mb to mitochondria, positively charged polar residues (Lys and Arg surrounding the heme cavity) (35) of oxy-Mb interact nonspecifically with the negatively charged anionic groups of phospholipids (the heads) of the outer mitochondrial membrane (OMM) (138, 139). At this level, there is a significant contribution of coulombic electrostatic forces, which may play an important role in the formation of the oxy-Mb/mitochondrial complex (138, 140).

MD simulation studies revealed the preferential location of Mb toward the heterogeneous mitochondrial surface and the structural arrangement of the Mb bound to the mitochondrial OMM (141). Mb “prefers” to bind the mitochondrial OMM where lipid composition of the OMM is characteristic to the contact regions with the cristae of the inner mitochondrial membrane (IMM) (141), where phosphatidylethanolamine (PE) molecules are bound to electron transport system complexes (I to IV) and likely facilitate conformational changes in the mitochondrial membrane (142–145). Furthermore, the contact stability and variability in Mb orientation toward OMM indicated that the most stable Mb orientations were with the heme crevice located close to the OMM, providing a narrow solute layer between Mb and OMM that could mediate the release of O_2_ from Mb into mitochondria (141). Although the lipid membrane was designed to mimic the mitochondrial membrane, it is highly challenging to incorporate all the integral and peripheral membrane-bound proteins into the membrane with the available in silico methods. Because of these limitations in MD simulations, binding predictions may differ when studied in in vivo physiological conditions. Fluorescence quenching studies revealed no specific complex formation between Mb and electron transport proteins of the inner mitochondrial membrane (IMM) or the proteins of the outer membrane (138). Despite these findings, recently a localization of Mb to the IMM as well as the OMM was observed with elegant digestion techniques (21, 146). The affinity of met-Mb toward the mitochondrial membrane was reportedly ∼1.5-fold higher than the affinity of oxy-Mb and remained unchanged in marginally acidic and basic conditions (pH 6–8) (TABLE 1) (140).

**Table 1.** Effect of native and artificial mitochondrial lipids on Mb affinity and O_2_ dissociation

Ligand Form of Mb	Binding Constant *(K*_m_,), µM^−1^			
	1,8-ANS		M540	
	pH 6.4	pH 7.4	pH 6.4	pH 7.4
Oxy-Mb	27 ± 04	23 ± 03	28 ± 03	25 ± 03
Met-Mb	38 ± 04	35 ± 04	32 ± 04	31 ± 04

	Fold Increase in O_2_ Dissociation in Presence of Lipids			
	Without liposomes	Lecithin	POPG	
Oxy-Mb	1	25	174	

	O_2_ Uptake by Mitochondria, µM min^−1^			
	Native, coupled	FCCP, uncoupled	Frozen	
Oxy-Mb	14.7 ± 1.5	83 ± 8	45.5 ± 5	
Oxy-Mb deoxygenation	15.6 ± 15	77 ± 8	46 ± 5	

Fluorescence quenching of mitochondria-bound lipid probes [1,8-ANS (1-anilinonaphthalene-8-sulfonate) and M540 (5-[3-γ-sulfopropyl-2(3H)-benoxazolylidene)-2-butenylidene]-1,3-dibutyl-2-thiobarbituric acid] by oxygenated myoglobin (oxy-Mb) and metmyoglobin (met-Mb) at pH 6–8. The ionic strength dependence of the myoglobin binding constants (*K*_m_) indicates the important role of coulombic electrostatics in interaction of myoglobin with artificial and natural mitochondrial membranes. The effect of liposomes on O_2_ dissociation from oxy-Mb in presence of neutral lecithin or negatively charged 1-palmitoyl-2-oleylphosphatidylglycerol (POPG) and O_2_ uptake from mitochondrial respiratory activity are shown. FCCP, carbonyl cyanide *p*-trifluoromethoxyphenylhydrazone. Data adapted from Refs. 138, 147, 148.

Unlike conventional hypotheses of O_2_ delivery by Mb (e.g., “oxygen depot” and “facilitated diffusion”), direct interaction of oxy-Mb with the OMM could substantially reduce oxy-Mb affinity toward the bound O_2_ ligand. Experimental results have shown that the interaction of Mb with the OMM leads to a conformational change in the heme cavity of Mb (134), which in turn results in a decrease of Mb affinity for O_2_ (and increased P_50_), thus facilitating O_2_ detachment from oxy-Mb even at normoxic Po_2_ (147).

Notably, mitochondrial respiration in rat liver was suppressed by ∼70% even with a surplus supply of buffer O_2_ in the presence of CO-bound Mb (132). Mb deoxygenation in cells and O_2_ transport to mitochondria was recently reported in natural and artificial bilayer phospholipid membranes (140, 148, 149). The effect of MbO_2_ autooxidation rate was found to increase during the interaction of Mb with phospholipid membranes and was significantly greater with negatively charged liposomes prepared with 1-palmitoyl-2-oleylphosphatidylglycerol (POPG) (TABLE 1). These congregated results confirmed that *1*) the rate of oxy-Mb deoxygenation is determined by the rate of mitochondrial respiration, *2*) deoxygenation relates to nonspecific binding of oxy-Mb to OMM phospholipids, *3*) O_2_ uptake by mitochondria is independent of the uptake of Mb-bound O_2_, *4*) electrostatic forces significantly contribute to Mb-membrane interactions, *5*) conformational changes of Mb near the heme pocket occur without altering protein secondary structure, and *6*) mitochondrial membrane interaction decreases the affinity of Mb for O_2_ by the shift in the oxy-Mb/Mb equilibrium toward the gas ligand-free protein.

From these observations related to Mb-mitochondrial interactions and Mb deoxygenation patterns, plus the established importance of blood Hb in body-wide O_2_ delivery, it is logical to assume that Mb is central to O_2_ delivery to mitochondria in support of muscle or heart work in lower-O_2_ partial pressure conditions (150). Extensive investigations of hypoxia-associated clinical phenotypes have revealed mitochondrial defects, concurrent dysregulation of ROS homeostasis, and cell death (151–162). Prolonged exposure to hypoxia prompts cells to activate adaptive mechanisms, particularly the overexpression of hypoxia-inducible factor 1α (HIF-1α), initiating significant alterations in mitochondrial structure, function, and dynamics (163). This sequence of events results in the impairment of mitochondrial fusion, depolarization, loss of mitochondrial DNA (mtDNA), and disruptions in respiration rate, accompanied by the distorted distribution of mitochondria within cells (164–166). Furthermore, investigations involving rat hearts have demonstrated a profound reduction in mitochondrial contact sites following acute ischemia, with immediate functional recovery observed upon reperfusion (167). In these circumstances, we presume that oxy-Mb may not establish a stable interaction with the OMM, necessitating further investigation.

However, at higher-O_2_ partial pressure conditions, the importance of Mb as an O_2_ carrier to sustain ongoing oxidative phosphorylation is debatable. For instance, overexpression of Mb in cultured murine C2C12 myotubes increased cellular O_2_ consumption by ∼50%, interpreted by the authors to mean that Mb is an integral part of the mitochondrial respiratory machinery (168). This is difficult to reconcile with the principle that mitochondrial O_2_ consumption is driven and regulated by chemiosmotic forces that link adenosine triphosphate (ATP) production to membrane electrochemical potential. An alternative interpretation to explain the data is that nonphysiological overexpression of Mb led to artifactual membrane damage and thus increased permeability of the inner mitochondrial membrane, hence driving enhanced O_2_ consumption to maintain electrochemical membrane potential. Although simple diffusion of dissolved O_2_ in cells is predominant and Mb provides a parallel path for intracellular O_2_ diffusion to support mitochondrial activity (31, 169, 170), other studies (171, 172) have raised doubts about Mb’s contribution to O_2_ overall flux, i.e., during exercise. Relevant questions with respect to Mb-mediated oxidative phosphorylation in humans may be considered:

- Is the process of repetitive steps of high-rate constant O_2_ off-loading at the Mb-mitochondrion interface, followed by Mb diffusion and loading with O_2_ at other locations, rapid enough to support the Mb’s proposed storage and release function in working muscle and heart?
- If Mb is critical to support oxidative phosphorylation as an O_2_ delivery protein, then why does its expression not change after exercise training regimens that elicit increases in tissue mitochondrial oxidative capacity and a higher maximal oxygen consumption (V̇o_2max_) in humans? A consistent finding over four decades of reports from human studies is that under normoxic environmental conditions training elicits significant increases in enzyme markers of mitochondrial oxidative capacity in skeletal muscle with no change in Mb protein levels (135, 136, 173–175) or Mb transcript abundance (176–179). Furthermore, muscle Mb protein abundance did not differ when untrained and trained individuals were compared (135, 180).

Nevertheless, delving into the intricate realm of Mb expression demands a comprehensive exploration of various physiological conditions. For instance, in the context of elite athletes undergoing exercise training at high altitudes, a discernible increase in skeletal muscle Mb concentration, oxidative enzyme activity, and endurance capacity has been reported (181), emphasizing the adaptability of Mb to challenging environmental conditions. Investigations into Mb expression extend to animal models, as well, i.e., in rabbits an increase in skeletal muscle Mb can occur in response to chronic contractile activity (25, 182). Also, very high Mb concentrations in the skeletal muscles of diving mammals and birds indicates that Mb could be a significant reservoir of accessible O_2_ availability in these species as they maintain function during breath holding (34, 36). The contrasting observations underscore the need for a deeper understanding of Mb’s multifaceted functions, including those beyond its potential role in O_2_ delivery to support oxidative phosphorylation.

## 4. COMPENSATORY MECHANISMS FOR CARDIOMUSCULAR METABOLISM IN MICE WITHOUT Mb

Oxidative metabolism of FAs and glucose (GLU) are central to support heart and muscle function. In the case of the beating heart, cycles of higher and lower O_2_ consumption and tissue Po_2_ are evident, and Mb-derived O_2_ has been hypothesized to contribute to pulses in mitochondrial respiration as Po_2_ ebbs and flows (147). Several groups have studied the functional consequences of Mb deficiency in heart and muscle through Mb knockout (Mb^−/−^) strategies. Mb^−/−^ mice are viable, fertile, and without any obvious signs of functional limitations (67, 68, 71). In one study, however, ∼32% of Mb^−/−^ embryos did not survive in utero at midgestation [embryonic day (E)9.5 and E10.5] because of lethal cardiovascular defects including myocardial thinning, prominent pericardial effusion, vascular insufficiency with peripheral hemorrhage, and reduced heart size (183). One perspective is that compensatory adaptations in the Mb^−/−^ mouse (i.e., increased capillary density and hematocrit) ensure that sufficient O_2_ delivery is maintained to support mitochondrial respiration and reduce the diffusion path length for O_2_ between capillary and the mitochondria (13, 67, 183–185). It was estimated that a 5% increase in diastolic perfusion rate, or a 4% increase in hematocrit, or a 16% increase in capillary density can functionally offset the loss of Mb in Mb^−/−^ mice (184).

In one study, compared to wild-type (WT) mice the Mb^−/−^ mice showed an increase in coronary flow (+24%), coronary reserve (+18%), and Hb concentration (+7%) (13). In another study, a significant increase in hematocrit level (+44%) was recorded in hypoxic Mb^−/−^ mice (186). Most studies of heart and cardiovascular outcomes in vivo or using perfused organ preparations have indicated that heart function is maintained in Mb^−/−^ mice, with certain caveats. Under normoxic conditions, aspects such as heart rate, blood pressure, left ventricular developed pressure (LVDP), the change in left ventricular pressure increase during systole per time (dP/d*t*), cardiac output, systolic and diastolic volumes, pressure-volume loops, myocardial O_2_ consumption, phosphocreatine and ATP, and heart size appeared normal in Mb^−/−^ mice (13, 31, 69, 70, 183, 186–189). However, one report indicated abnormalities in left ventricular mass (higher), ejection fraction and stroke volume (lower), and several other parameters in Mb^−/−^ mice (190). Merx et al. (191) also reported lower cardiac function indexes as measured by echocardiograph and isolated heart preparations from Mb^−/−^ mice. We interpret these collective findings to indicate that loss of Mb can alter heart function in sometimes subtle ways, but these events occur in the presence of a maintained mitochondrial oxidative metabolism to sustain the energy needs of cardiac tissue.

Data regarding skeletal muscle performance effects in Mb^−/−^ mice are mixed. In one study, Mb^−/−^ mice demonstrated normal exercise capacity in a treadmill paradigm (4 days, ∼36 min per day) and after assessment of endurance performance at an intensity of exercise requiring high rates of O_2_ flux (68). In that study, there were no distinct histological changes in muscle tissue parameters. Furthermore, no significant changes were observed in mitochondrial content, vascular supply, or myosin isoform expression compared to WT mice (68). In a different study using a treadmill model in a separate line of knockouts, Mb^−/−^ mice displayed more rapid onset fatigue (by ∼6 min) and lower distance run (by ∼120 m), with decreased heart contractility, reduced heart rate, decreased cardiac output, and impaired systolic shortening (191). Fatigue was indistinguishable between WT and Mb^−/−^ when following ex vivo stimulation of extensor digitorum longus or soleus (100 Hz) in normoxic or hypoxic conditions (68), but soleus force was eventually lowered by ∼12% in Mb^−/−^ muscle preparations in a normoxic 60-min protocol at a slower frequency (40 Hz) (71). In a recent paper, we reported that day-to-day ambulatory activity remained normal in Mb^−/−^ mice (albeit slightly higher in knockout males and slightly lower in females) (67). In that study, no evidence for a fiber type transition in muscles of Mb^−/−^ mice was observed, but the ratio of number of capillaries to fibers was significantly increased in gastrocnemius and soleus tissues of Mb^−/−^ mice (67). Additionally, in a recent study, compared to WT mice Mb^−/−^ mice had lower body mass, heart, and hind limb muscle mass with significantly reduced maximum running exercise performance in closed one-lane motorized treadmill experiments (192). Thus, literature reports for Mb knockout impacts on performance (and activities requiring muscle work) have been mixed, and when an effect was present it was generally modest.

With respect to tissue fuel metabolism, in male Mb^−/−^ mice GLU uptake and catabolism in heart was increased (70, 183) and heart membrane glucose transporter type 4 (GLUT4) protein was ∼60% higher (70). Palmitate (PLM) carbon flux to tricarboxylic acid-generated glutamate in the heart was reduced in Mb^−/−^ mice in one case (70), but PLM flux to acetyl-CoA was unchanged in a different study (183). Thus, in the absence of Mb, cardiac GLU metabolism appears to be increased but effects on cardiac fatty acid oxidation (FAO) are not clear. Interestingly, whole body shifts in fuel utilization are not apparent in male or female Mb^−/−^ mice, since the respiratory exchange ratio [aka respiratory quotient (RQ), which shifts in response to relative oxidation rates of carbohydrates, proteins, and fats] did not differ compared to WT mice under a variety of conditions including high-fat feeding and cold exposure that were designed to enhance FAO (67). In that same study, blood GLU (postabsorptive or after a GLU tolerance test or insulin tolerance test) and postabsorptive blood FAs also were not altered by the absence of Mb.

In another study, cardiac function in Antarctic fishes with varying O_2_-binding protein expression (Hb and Mb) were investigated (193). Three species, *Gobionotothen gibberifrons* (expresses Hb and Mb), *Chionodraco rastrospinosus* (lacks Hb but expresses Mb), and *Chaenocephalus aceratus* (lacks both Hb and Mb) showed distinct mitochondrial morphologies and volume to surface area. Antarctic fishes lacking Mb in cardiac muscles maintain O_2_ flux by increasing mitochondrial densities, aided by a decreased diffusion distance between ventricular and mitochondrial membrane (193). In the same study, high mitochondrial densities resulted in high intracellular lipid content, which may enhance O_2_ diffusion. Despite these ultrastructural variations, oxidative capacities were found to be similar among these species, indicating consistent aerobic metabolic capacity (193). In another study, the Antarctic icefish family evolutionally lack cardiac Mb expression and are associated with pale heart colors and low metabolic rate, but paradoxically this species can migrate long distances (194, 195). This complete loss of Mb-assisted O_2_ delivery to heart muscle is thought to be a common facet of teleost biodiversity.

We conclude that the generally preserved cardiac function, aerobic exercise, myotube contraction phenotypes, and ambulatory activity under normoxic conditions in Mb^−/−^ mice and in species lacking significant Mb argue against Mb being necessary and sufficient to sustain oxidative phosphorylation for ATP production. This conclusion is further supported by a lack of difference in whole body energy expenditure as estimated from indirect calorimetry, comparing male and female Mb^−/−^ to WT mice (67).

## 5. “ALTERNATIVE” FUNCTIONS OF Mb

### 5.1. Mb as a Regulator of Nitric Oxide Pools

Nitric oxide (NO) is a well-known mediator of many physiological functions, viz. neurotransmission, platelet aggregation, macrophage function, vasodilation, etc. (196). Because of vasodilating properties, NO regulates blood flow to tissues and thus helps support supply of blood-derived respiratory substrates to mitochondria (197, 198). Skeletal muscle is the major nitrate ($NO_{3}^{-}$) reservoir (∼226 nmol/g) in the human body (199), and factors such as exercise enhance the Mb-mediated NOD activity in muscle (i.e., degradation of NO by oxy-Mb to form met-Mb and $NO_{3}^{-}$) (200). NO serves as a regulator not only of respiration but also of ROS generation (201). By interacting with CcOX, NO has the capacity to enhance the production of superoxide by the respiratory chain (202). Consequently, the NO-dependent mitigation of respiratory inhibition may contribute to a reduction in mitochondrial ROS generation, potentially mitigating oxidative stress and influencing redox signaling in both the heart and skeletal muscle.

NO signals mainly engage three mechanisms: *1*) NO triggers the activation of soluble guanylate cyclase, leading to the conversion of guanosine 5′-triphosphate into 3′,5′-cyclic guanosine monophosphate, with the quantity produced directly correlating with the concentration of NO; *2*) NO readily and reversibly establishes covalent bonds with reactive cysteine thiols in diverse target proteins, forming S-nitrosothiol groups; and *3*) NO promotes mitogen-activated protein kinases through intracellular formation of peroxynitrite (46). NO signaling in skeletal muscle is implicated in the control of multiple functions, including muscle metabolism, excitation-contraction coupling and contractility, immune cell signaling, cell growth, and neurotransmission (203). NO also may regulate mitochondrial function by binding to CcOX, the terminal enzyme in the electron transport chain (198, 204), to inhibit the ubiquinone-cytochrome *b* region. The latter led to increased superoxide radical production (202) in rat heart mitochondria and submitochondrial particles.

Besides binding O_2_ and CO, under normoxic conditions Mb effectively binds (205) and scavenges (45) NO. Mb might play a significant role in scavenging NO from the cell in a process by which oxy-Mb converts to met-Mb and produces $NO_{3}^{-}$ (200). Regeneration of oxy-Mb from met-Mb by met-Mb reductase enzyme helps make oxy-Mb readily available for another NO degradation cycle, effectively reducing cytosolic NO concentration (206). To compare NO deoxygenation mechanisms with oxy-Mb, computational studies using truncated Hb from *Mycobacterium tuberculosis* revealed that at the diatomic NO enters the distal portion of the heme pocket, and in the second step it rapidly converts the noncovalently Fe-bound NO into Fe^2+^-N = O or with Fe^2+−^O-O^δ−^ to produce Fe^3+^-OH_2_ and $NO_{3}^{-}$ (207). The rate constants of NO binding to deoxy-Mb and NO-induced oxidation of oxy-Mb are similar (*k*′_ox,NO_ = 30–50 µM^−1^/s) (47) but significantly elevated when His-E7 is mutated with apolar amino acid residues like H64A, H64V, and H64L in deoxy-Mb (208–210). With NO being a key mediator of diverse metabolic functions, these actions of Mb may provide a physiological strategy for maintaining NO homeostasis through its NO scavenging role. This NO homeostasis cycle plays an important role in determining the dose-response curve of the NO effects on coronary blood flow and cardiac contractility (200). Furthermore, additional studies have shown that Mb’s scavenging capability extends beyond NO to include ROS, introducing a novel antioxidant defense mechanism (189).

Prompted by observations in the Hb literature, an O_2_-sensitive system of NO production from $NO_{2}^{-}$ by deoxy-Mb was hypothesized (211, 212). This was validated with purified horse Mb and murine heart preparations in which O_2_ saturation was titrated downward from 45% to 5% or exposure to $NO_{2}^{-}$ was increased during hypoxia (212). In that study, NO-associated depression of oxidative phosphorylation in perfused hearts (presumably due to inhibition of CcOX) was evident in WT but not Mb^−/−^ mice, and NO production from $NO_{2}^{-}$ was reduced by 60% in Mb^−/−^ heart homogenates. Similarly, the $NO_{2}^{-}$ and $NO_{3}^{-}$ levels were significantly reduced in the heart muscles of Mb^−/−^ mice at rest (192). However, in locomotory muscles, Mb^−/−^ mice had significantly lower $NO_{2}^{-}$ content at rest compared to WT mice, whereas there were no differences in resting $NO_{3}^{-}$ content between WT and Mb^−/−^ mice (192). Furthermore, applied strenuous exercise, running until exhaustion, resulted in a similar pattern of changes in $NO_{2}^{-}$ and $NO_{3}^{-}$ levels in the muscles and no changes in activities of NO^•^ synthase (192).

In another study, respiration of isolated rat liver mitochondria was reduced in the presence of equine heart deoxy-Mb plus $NO_{2}^{-}$, a combination that led to NO production (211). Under conditions of low Po_2_, deoxy-Mb undergoes a transition from being a scavenger of NO to functioning as a NO producer. This shift occurs as the partial pressure of inspired oxygen ($PI_{O_{2}}$) approaches the P_50_ for O_2_ binding to Mb, ∼3 Torr. Within this range of Po_2_, skeletal muscles exhibit the ability to convert $NO_{3}^{-}$ to $NO_{2}^{-}$ through the activity of resident enzymes belonging to the xanthine oxidoreductase (XOR) family. In each of these above two studies (211, 212), NO production in response to hypoxia or $NO_{2}^{-}$ was unaffected by xanthine oxidoreductase (XOR) inhibitor, suggesting that effects were largely through deoxy-Mb (although other hypoxia-triggered NO sources beyond XOR could not be ruled out). Highlighting further the potential for Mb to impact NO balance as an NO producer, cardiomyocytes from Mb^−/−^ mouse hearts showed impaired $NO_{2}^{-}$-dependent NO generation, lower nitrosylation, and reduced production of cyclic guanosine monophosphate (cGMP, an essential signaling agent in vasodilation) (45).

It has been proposed that Mb-generated NO signaling closely regulates mitochondrial energetics and thus adjusts oxidative metabolism downward (via NO inhibition of the electron transport chain) in response to a reduced O_2_ supply (211–213). This was suggested by studies in mice, where NO generated by $NO_{2}^{-}$ reduction inhibited cellular respiration after cardiac injury (213). An improved recovery against myocardial infarction in WT compared to Mb^−/−^ mice clearly suggests a functional role for Mb in regulating cellular hypoxia responses (213). In another study the $NO_{2}^{-}$ levels in the skeletal muscles of Mb^−/−^ and WT mice were nearly similar and they showed lower $NO_{2}^{-}$ concentrations after diet supplementation with the NOS inhibitor *N*^G^-nitro-l-arginine methyl ester (l-NAME) (48). However, expression of proteins that are related closely to NO metabolism, like XOR and sialin (a $NO_{2}^{-}$ transporter), were upregulated nearly 85% and 89% in the Mb^−/−^ skeletal muscle from that study, as if these systems were adapting to a loss of Mb-directed NO production.

In conclusion, biochemical and tissue-level studies support the idea that Mb is an important regulator of cellular NO pools, through O_2_-dependent mechanisms in which Mb is a NO scavenger during normoxia but functions as a NO producer (and thus possibly dampening the electron transport chain) as Po_2_ drops. These dynamics may enable myocytes and cardiomyocytes to have a “governor” system to blend both oxidative and nonoxidative metabolism depending upon O_2_ availability, a concept put forth in principle by Shiva et al. (211) and Rassaf et al. (212) and further elaborated by Clanton (206) for working muscle. These concepts require further validation in terms of physiological relevance, in light of Mb^−/−^ mouse studies in which heart and muscle function are generally preserved and energy expenditure remains normal. It is also important to note that in working muscle and heart (in which Po_2_ drops) mitochondria consume high amounts of O_2_ in support of oxidative phosphorylation, further raising questions about the NO-electron transport chain inhibition concept in situ. As noted previously (211) and reviewed recently (214), other globins such as Cygb could contribute to NO homeostasis alongside Mb, making interpretations from Mb^−/−^ models more difficult. Regardless of specifics, the extant literature strongly supports a physiological role for Mb in regulation of tissue NO homeostasis. As discussed below, Mb’s regulation of NO pools may contribute to its function as a Po_2_-sensitive regulator of cellular signaling pathways.

### 5.2. Metabolic Regulation in Brown Adipose Tissue and Adipocytes: Mb as a “Metabolic Sensor” That Regulates Cellular Signaling Pathways and Gene Expression

Most studies of Mb function have focused on heart and muscle or have studied Mb in the context of biochemistry experiments using purified Mb preparations. As mentioned in previous sections, the potential impact of Mb on whole body energetics and physiology remains open to debate. Also notable is the fact that most Mb^−/−^ mouse studies have focused on males. Recently, we challenged male and female WT and Mb^−/−^ mice with high-fat diet (45% of calories) for ∼13 wk and tested responses to metabolic challenges such as cold exposure and GLU and insulin tolerance tests (67). Mb^−/−^ mice showed no differences in respiratory exchange ratio (RER, a measure of tissue fuel choice), energy expenditure (EE) as estimated by indirect calorimetry, or GLU or insulin tolerance. Interestingly, however, Mb^−/−^ female mice showed an average of ∼42–48% more body fat accumulation compared to WT females, despite no evidence for altered food intake (67). These observations suggested that Mb is involved in systems regulating bioenergetics and “metabolic efficiency” (i.e., conversion of fuel energy to ATP) and that some physiological actions of Mb are impacted by sexual dimorphism. In addition, we (67) and others (21) did not observe differences in whole body RER in Mb^−/−^ mice, even when challenged by cold exposure, which promotes fat combustion.

**FIGURE 5.**
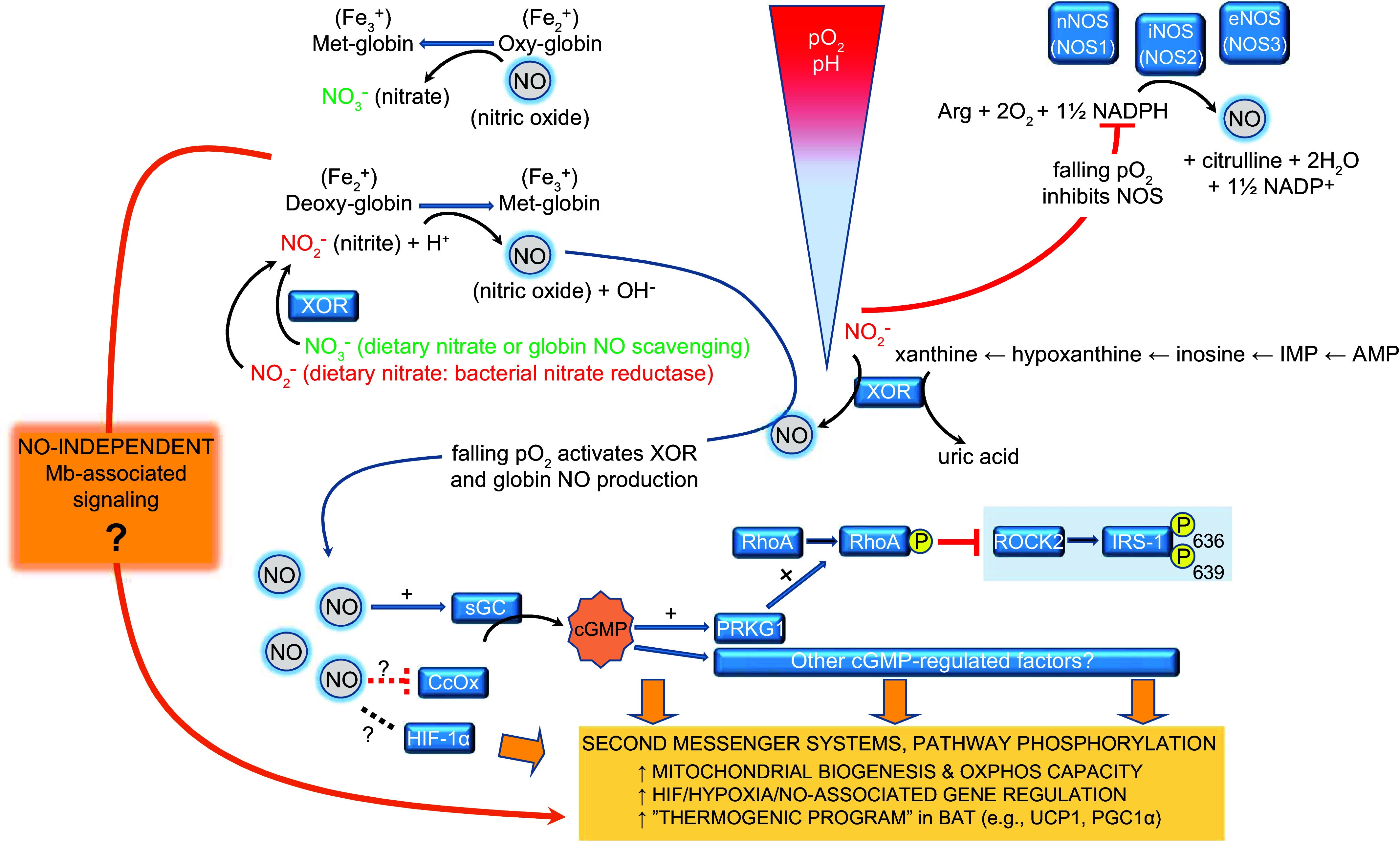
Conceptual model of Po_2_-dependent globin/myoglobin (Mb) function as a nitric oxide (NO)-regulating “O_2_ sensor” that modifies gene expression and molecular pathways: illustration for brown adipose tissue (BAT). In this model, an important role for Mb is to modify intracellular signaling cascades to regulate processes such as gene expression, protein phosphorylation, and other pathways to regulate metabolism, responses to hypoxia, or other adaptive functions (in this case, to sustain BAT thermogenic activation). It is hypothesized that, in addition to regulation by Po_2_, metabolite binding, pH, and proximity to membrane sites could alter the signaling properties of Mb. CcOX, cytochrome *c* oxidase; eNOS, endothelial nitric oxide synthase; iNOS, inducible nitric oxide synthase; nNOS, neuronal nitric oxide synthase; sGC, soluble guanylate cyclase; XOR, xanthine oxidoreductase. Image reproduced from Ref. 19, with permission from the American Physiological Society.

Brown adipose tissue (BAT) is characterized by a higher capillary density and mitochondrial O_2_ consumption and in energy homeostasis by generating heat from chemical energy via uncoupled oxidative phosphorylation (215). Mb transcript and protein expression in mouse BAT is increased during brown adipogenesis (19) or cold exposure in mice (216, 217) and rats (218). Thus, one may ask, “Does Mb in BAT have a physiological role, or is expression vestigial in nature since brown adipocytes and myocytes share a muscle cell lineage?” In light of increased adiposity and metabolic efficiency (especially in females) in Mb^−/−^ mice, and the well-established role for BAT in driving thermogenesis and uncoupled respiration, we considered whether BAT phenotypes differ in Mb^−/−^ mice (19). We first proposed a working model (19) that extended earlier concepts regarding Po_2_-dependent Mb-NO associations in heart and muscle (e.g., Refs. 206, 211, 212), keeping in mind the well-known regulation of brown adipocyte function via NO-related signals that are involved in “thermogenic activation” through upregulation of genes such as Ucp1 and PGC1α (219–223). This model proposes that oxy-Mb/deoxy-Mb “toggling” and intracellular NO management regulate downstream molecular signaling pathways, gene expression, and metabolic efficiency in BAT; these activities may also manifest in other Mb-expressing tissues (FIGURE 5). Important to note when considering this model is that “activated” thermogenic BAT is an avid O_2_ consumer and coincident with accelerated oxidative flux during thermogenesis, tissue Po_2_ drops (19, 206, 212, 224), analogous to working muscle.

Consistent with an important function for Mb in BAT, we observed that Mb mRNA and protein were upregulated in the course of murine brown adipocyte differentiation and maturation (19). In line with the working model of Mb as a metabolic regulatory protein, in the intrascapular BAT many transcripts significantly reduced in female and male Mb^−/−^ mice were NO and hypoxia associated (i.e., HIF1α associated) (19). Especially in females, there was a significantly higher prevalence of larger-sized fat droplets and ∼30% lower protein expression of the electron transport chain protein CcOX-IV (19). In addition, Perm1 gene expression was significantly reduced; in muscle this is a factor that participates in PGC1α-dependent mitochondrial biogenesis and increases in oxidative metabolism machinery. The phenotypes in Mb^−/−^ BAT suggested a reduced BAT oxidative capacity and dampened BAT thermogenic activity in the absence of Mb, especially in females, which we speculated may contribute to the sex-specific higher adiposity and BAT lipid droplet sizes in Mb^−/−^ versus WT mice (67).

Following our Mb^−/−^ reports on metabolic phenotypes and BAT, Aboouf et al. (20) reported that chow-fed female Mb^−/−^ mice have significantly increased adiposity. This group also observed lower interscapular BAT mitochondrial oxidative phosphorylation capacity, reduced mitochondrial DNA, reduced markers typical of thermogenically active BAT (e.g., UCP1, Cidea, PGC1α, etc.), and higher prevalence of large lipid droplets, especially in females. In addition, loss of Mb protein increased PLM levels of lipid droplets (20). In 2022, Christen et al. (21) further explored the potential role of Mb in BAT. The protein and mRNA levels of Mb were significantly induced during differentiation of immortalized murine brown adipocytes and were increased significantly after cold exposure in mice, with the changes in Mb mRNA correlated with UCP1 mRNA. Consistent with lower BAT CcOX-IV protein (19) and reduced BAT tissue oxidative respiration (20) in Mb^−/−^ mice, cultured brown adipocytes with Mb knockdown (∼50% lower Mb) or derived from Mb^−/−^ mice displayed lower overall O_2_ consumption in response to mitochondrial substrates in what appeared to be a gene dose pattern (21). The latter authors did not observe any differences in lipid droplet phenotype in cultured brown adipocytes from Mb^−/−^ mice or after siRNA Mb knockdown, with the interpretive caveat that cells were not grown with lipid substrate and primary brown adipocytes from Mb^−/−^ mice were from male donors only.

Altogether, these recent findings are consistent with Mb as an important player in support of BAT metabolism and thermogenesis and support the working model that an important role for Mb is to serve as a metabolic sensor that participates in molecular signaling pathway regulation, in part by alterations in cellular and subcellular NO pools (FIGURE 5). This perspective differs somewhat from previous models that focused on Mb-driven, direct NO inhibition of cytochrome *c* oxidase activity (206, 211, 212), but these multiple functions are not mutually exclusive. Although the exact mechanisms remain to be established, we suspect that Mb communicates Po_2_/NO status with other cell systems through protein-protein interactions and/or signaling cascades to modify cellular functions including gene expression. In support of this idea, as noted above, there was a set of transcripts related to NO/stress/HIF1-α-hypoxia pathways that were significantly reduced in the BAT of Mb^−/−^ mice (19), as were PGC1α (20) and a related factor, Perm1 (19). Interestingly, overexpression of human Mb in immortalized murine brown adipocytes led to increases in a variety of metabolically important genes and increased responses to β3 receptor activation (i.e., lipolysis, lactate, PKA phosphorylation); the latter were blunted in BAT or brown adipocytes lacking Mb (21).

**FIGURE 6.**
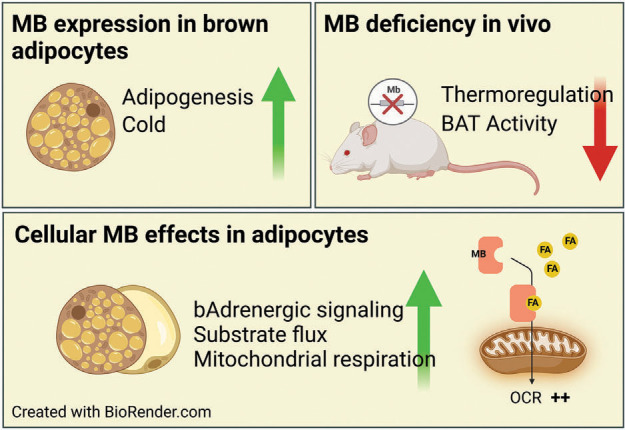
Model of thermogenic activation in brown adipose tissue (BAT) due in part to myoglobin (Mb)-mediated lipid shuttling in support of mitochondrial fatty acid (FA) oxidation. Consistent with abnormal BAT lipid droplet and other BAT phenotypes in Mb knockout mice as first described in Ref. 19 and then Ref. 20, Christen et al. (21) highlighted the participation of Mb in FA trafficking and BAT thermogenesis activation through analysis of the effects of gene knockout in vivo and in cell models. OCR, oxygen consumption rate. Image adapted from Ref. 21 from *Clinical and Translational Medicine* per CC-BY 4.0 Open Access.

In terms of regulation of Mb expression in BAT (i.e., in response to cold) (FIGURE 6), surprisingly, the direct activation of the adrenergic β3 receptor failed to induce, and actually repressed, Mb expression in brown adipocytes (21). Fournier et al. (217) discovered that Mb and PGC-1α expression were coincidentally induced in BAT tissues of male mice via inhibition of the myostatin/activin receptor IIB (ActRIIB) pathway or by cold exposure. By gene knockdown in immortalized brown adipocytes, reductions of the transcription factor nuclear respiratory factor 1 (NRF1) and/or its coregulator PGC1α profoundly blunted Mb mRNA and protein expression, supporting important and perhaps central roles for these factors in activating Mb expression.

In summary, there is strong and consistent evidence from mouse models that Mb in brown adipocytes has an important physiological role as a metabolic regulator impacting dynamic shifts in fuel trafficking, bioenergetics, and signaling cascades. Whether or not a similar metabolic regulator role manifests in other Mb-expressing tissues remains to be confirmed, and specific molecular mechanisms involved in Mb-associated cell signaling remain to be fully established. Connections between Mb, PGC-1α, NO, and related factors seem clear. However, the full suite of players in this nexus awaits discovery, and specific relationships are likely context dependent, i.e., involving sexual dimorphism, Po_2_, or temperature shifts. Furthermore, changes in the cellular metabolite milieu, Mb oxygenation, and pH, discussed in the following sections, could in theory influence the metabolic regulator role of Mb.

### 5.3. Mb Interactions with Cellular Metabolites

As a protein associated with tissue bioenergetics, O_2_/NO binding, and intermediary metabolism, it is reasonable to consider whether additional Mb-metabolite interactions occur and regulate Mb function. A growing body of literature indicates that Mb is a binding protein for a variety of small molecules including certain lipids and carbohydrates. These interactions may point to a role for Mb in trafficking and sequestration of metabolites in a way that regulates their actions and metabolism in the cell. In addition, it is interesting to consider whether the binding of metabolites to Mb can influence Mb activities including its roles in O_2_/NO management and ETC regulation or its functions as a cell signaling protein that regulates molecular pathways as described in previous sections.

#### 5.3.1. Mb-lipid binding.

LCFAs are a primary energy source supporting metabolic activities of type I myofibers and cardiomyocytes (225). LCFAs, especially saturated LCFAs and derivatives such as long-chain acylcarnitines, ceramides, or diacylglycerides (DAGs), have been implicated in promoting muscle insulin resistance and metabolic dysfunction, and the subcellular localization and sequestration pools can modify LCFA-associated lipotoxicity (226, 227). Management of the trafficking and sequestration of LCFAs is complex in vertebrates and other organisms. Several proteins bind LCFAs and are found in different compartments of cells and tissues: plasma compartment (serum albumin) (228); cytosolic compartment of mammalian cells [fatty acid-binding proteins (FABPs)] (229); cytosol of plant cells (nonspecific lipid-transfer protein) (230); nucleus of mammalian cells [i.e., bound to peroxisome proliferator-accepted receptor (PPAR) and hepatic nuclear factor 4] (231, 232); and bacterial membrane (halorhodopsin) (233). The underlying mechanisms by which LCFAs are trafficked in muscle cells and cardiomyocytes remain to be fully cataloged. Accumulating evidence points to Mb as a lipid-binding protein.

**FIGURE 7.**
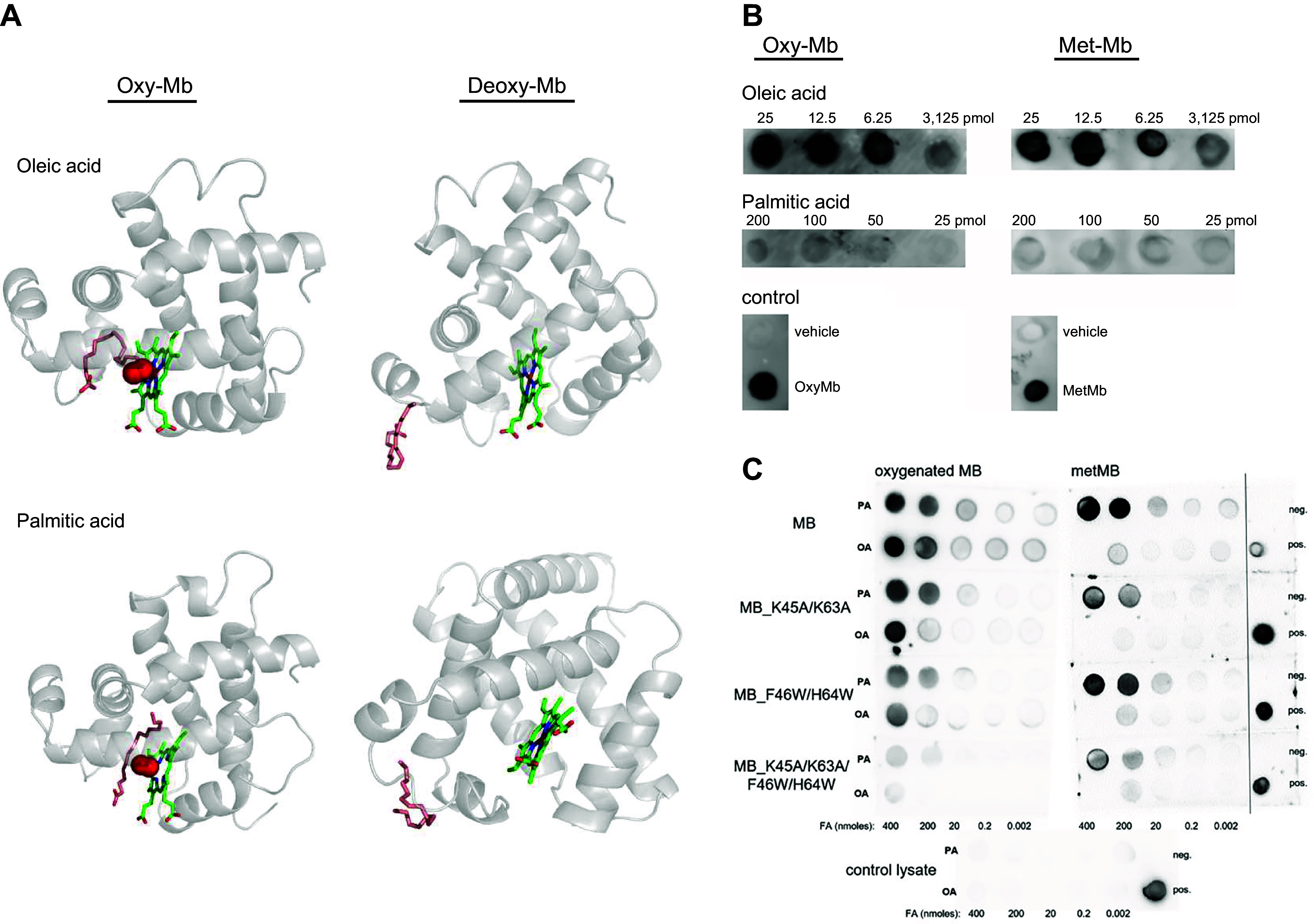
Oxygenated myoglobin (oxy-Mb) binds long-chain fatty acids (LCFAs). *A*: molecular dynamics (MD) simulation snapshots of murine oxy-Mb and deoxygenated myoglobin (deoxy-Mb) interacting with LCFAs [oleic acid (OLM) and palmitate (PLM)]. LCFAs avidly bind near the heme center and attain “U”-shaped configurations in the hydrophobic pocket of oxy-Mb. LCFAs do not show any specific binding with deoxy-Mb and randomly interact at different regions far away from the heme center. The protein backbone is represented as ribbon shape, whereas ligands, key side chains, and heme are represented as sticks. Oxygen molecules are in VdW representation (red spheres). Water molecules and ions are hidden for clarity. *B*: protein-lipid overlay assay using nitrocellulose membrane spotted with OLM and PLM and incubated with equine heart oxy-Mb as well as metmyoglobin (met-Mb). Image adapted from Ref. 190 from *Scientific Reports* per CC-BY 4.0 Open Access. The results clearly depict that OLM binds avidly to both oxy-Mb and met-Mb more than PLM. *C*: dot-blot lipid overlay assay using nitrocellulose membrane spotted with oxy-Mb and met-Mb and mutants and different concentrations of LCFAs. Reduced binding of LCFAs with the double Mb mutants (MB_ K45A/K63A and MB_F46W/H64W) and complete loss of fatty acid (FA) binding with the Mb mutant lacking all 4 residues (MB_K45A/F46W/K63A/H64W) were clearly demonstrated. Bound Mb detected with anti-Mb antibody. OA, oleic acid; PA, palmitic acid. Image adapted from Ref. 21 from *Clinical and Translational Medicine* per CC-BY 4.0 Open Access.

Freshly prepared rat heart oxy-Mb was found to interact with oleic acid (OLE), albeit at 40 times lower binding affinity than bovine serum albumin (BSA) (9). Freshly prepared Mb from chicken gizzard and heart showed higher binding affinity toward LCFAs in the oxy-Mb versus deoxy-Mb form (58). A growing body of evidence stemming from these original observations has confirmed oxy-Mb as a protein involved in binding FAs (18, 54–58). Numerous studies interrogated the pathways and mechanisms of movement of small-molecule ligands (e.g., O_2_ or noble gases) within the cavities and tunnels of Mb, but only limited studies have been performed on lipid binding pockets. Leveraging protein motif homologies to all known FABPs, computational predictions have shown that LCFAs (PLM and OLE) bind equine oxy-Mb in a binding pocket located in the hydrophobic cavity of the oxy-Mb structure in direct contact with the heme and wrapping around the bound O_2_ (FIGURE 7*A*) (18). However, short- to medium-chain FAs were unable to bind to both oxy- and deoxy-Mb (16). Consistent with computational results, experimental data employing nuclear magnetic resonance (NMR) measurements with horse Mb clearly demonstrated that LCFAs specifically bind to oxy-Mb or MbCO and nonspecifically to Fe^3+^ MbCN (a structure-function model of the ligated physiological state of Mb), while it does not interact with deoxy-Mb (53, 56, 234, 235).

However, with the use of anti-Mb antibody blots to immobilize Mb, there was evidence of LCFAs (e.g., PLM and OLE) binding with equine heart met-Mb, albeit at lower levels than oxy-Mb, at least for OLE (FIGURE 7*B*) (190). Consistent with the latter, more recently this method suggested some retained met-Mb binding of PLM but not OLE to a human Mb preparation (FIGURE 7*C*) (21). The results suggest that there might be nonspecific binding regions irrespective of the specific binding at the heme center (i.e., in other hydrophobic crevices that serve as conduits for the gaseous ligands), a prospect discussed below. By drawing upon mutational data related to ligand movement in the Mb protein, as outlined in sect. 2.2, we predict that the Tyr(B10) Mb mutant would have a significant effect exhibiting varied affinities for lipid binding. This variability is expected to arise from its unique O_2_ affinities toward Mb, subsequently influencing the levels of oxy-Mb within the cell. Binding studies using isothermal titration calorimetry (ITC) of solutions enriched in oxy-Mb versus met-Mb coincident with LCFAs revealed specific binding characteristics only with oxy-Mb (18). Similar to serum albumin (228, 236–241), Mb also has higher affinity [*K*_d_(app)] toward unsaturated FAs (∼6.1 µM for OLE) than saturated FAs (∼29 µM for PLM) (TABLE 2) (18). However, comparing FA binding to albumin and FABPs, high *K*_d_ values indicate that lipid metabolites bind with lower affinity to oxy-Mb, and further research is needed to assess the relative contributions of specific versus nonspecific binding of lipids to Mb.

**Table 2.** Dissociation constant values of lipids to proteins

	Fatty Acids				Acylcarnitines		
	C12:0	C16:0	C18:1	C18:2	C12:0	C16:0	C18:1
Oxy-Mb	509	29.1	6.17	ND	864	9.76	1.98
Met-Mb							
Human plasma albumin	0.41	0.016	0.0038	0.012			
Bovine serum albumin	0.07		0.008	0.002			
Bovine heart FABP		0.004	0.005	0.029			
Human adipose FABP		0.083	0.058	0.092			
Murine adipose FABP		0.077	0.056	0.102			
Rat heart FABP		0.014	0.01	0.032			
Rat intestine FABP		0.03	0.039	0.14			
Rat liver FABP		0.023	0.009	0.029			
Human heart-type FABP	0.2	0.14	0.29				2.21

Values are dissociation constants (*K*_d_) in µM. For comparing with myoglobin (Mb), only 1:1 binding ratio with the selected lipids are given here with all the lipid binding proteins. All fatty acid binding proteins (FABPs) listed here are recombinant proteins. Met-Mb, metmyoglobin; ND, not determined; oxy-Mb, oxygenated myoglobin. Data adapted from Refs. 18, 228, 239, 242, 243.

**FIGURE 8.**
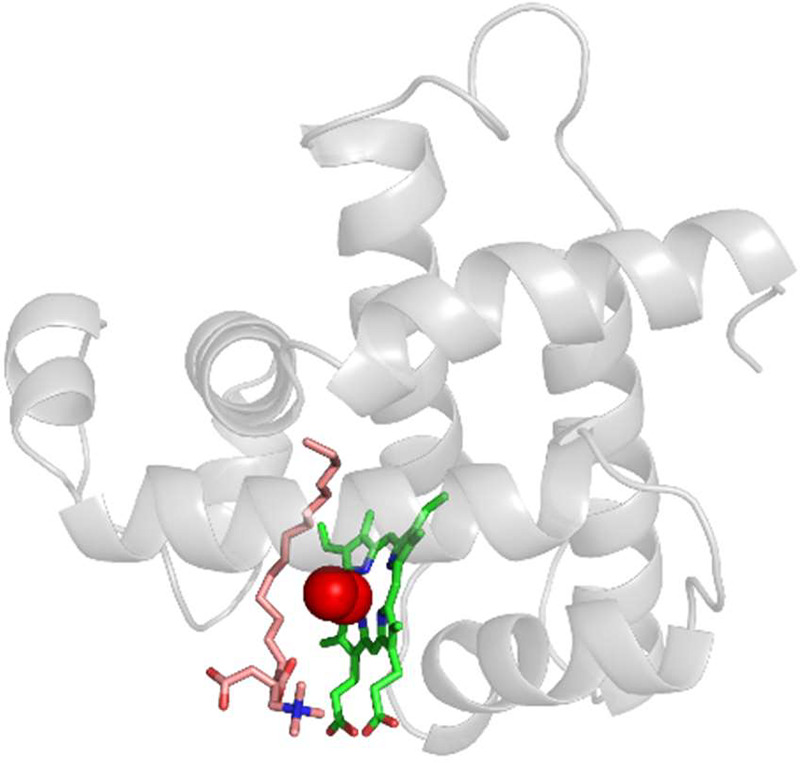
Oxygenated myoglobin (oxy-Mb) with palmitoylcarnitine. Snapshots of murine oxy-Mb simulated with palmitoylcarnitine. The protein backbone is represented as ribbon shape (gray), whereas ligands (magenta) and heme (green) are represented as sticks. Oxygen molecules (red) are shown in spheres. Water molecules and ions are hidden for clarity.

Long-chain acylcarnitines (LCACS) are derivatives of fatty acyl-CoAs converted by carnitine acyltransferases (e.g., the carnitine palmitoyltransferases) at the mitochondrial surface or facing the matrix. Since LCFAs were shown to bind oxy-Mb, LCACs were also hypothesized to bind oxy-Mb (18) (FIGURE 8).

In support of more avid binding of oxy-Mb with LCACs, ITC thermodynamic binding studies showed significantly lower *K*_d_ values with the LCACs in comparison to their corresponding LCFAs (TABLE 2) (18). In addition to O_2_-dependent specific binding to Mb, recent models suggest nonspecific binding, consistent with biochemistry studies described previously for LCFA (190).

#### 5.3.2. Mb’s potential role in fatty acid trafficking and lipotoxicity.

LCACs are markers of incomplete β-oxidation and serve as natural zwitterions that modify membrane-associated systems, which may contribute to FA-associated insulin resistance, inflammation, and myocyte stress responses related to cardiac ischemia (244–247). We have suggested that oxy-Mb-LCFA and oxy-Mb-LCAC binding is protective against lipotoxicity (18), an idea supported by results reported by others (190). Cardiac ischemia regions are by definition sites in which deoxy-Mb would be prevalent and LCFA/LCAC binding and sequestration would be negligible. Interestingly, sites of cardiac ischemia damage have very high levels of LCAC, and the latter were implicated in driving functional deterioration of heart mitochondria (248). Thus, it is reasonable to consider that in low-Po_2_ situations such as cardiac ischemia Mb off-loading of LCACs and LCFAs would occur as oxy-Mb transitions to deoxy-Mb, in turn increasing “free” lipids that may then contribute to lipotoxic outcomes. In a related perspective, the altered lipid droplet phenotypes in Mb^−/−^ BAT (19, 20) and cardiomyocytes (247) suggest that the LCFA binding to Mb participates in intracellular lipid storage. As discussed in Aboouf et al. (20) and elaborated more fully in the experiments conducted by Armbruster et al. (249) that tracked BODIPY FL C16 in MDA-MB468 and MCF7 cell lines, there is evidence that oxy-Mb helps retain LCFA in “cytosolic” areas of the cell whereas under hypoxic conditions (dominated by deoxy-Mb) the LCFA are increasingly relegated to a lipid droplet fraction. The authors hypothesized that greater lipid droplet formation with deoxy-Mb off-loading of LCFAs under hypoxic conditions would protect against lipotoxicity, a perspective that contrasts with the notion of lipid off-loading promoting the “free” pools of lipotoxic LCFAs and LCACs (18). Perhaps both events are not mutually exclusive: despite newly released lipotoxins eliciting cell stress responses during hypoxia, the full effects of the metabolites might be mitigated to varying degrees through lipid droplet storage.

Chemical reactions involving lipid oxidation are well established (250), but mechanisms of Mb-induced oxidation/peroxidation of polyunsaturated FAs (PUFAs) are still a matter of dispute (63, 64). Spontaneous lipid radical formation or direct reaction of PUFAs with molecular O_2_ is thermodynamically unfavorable. Some of the consequences of uncontrolled oxidative stress (imbalance between the prooxidant and antioxidant levels) are cell, tissue, and organ injury caused by oxidative damage. Lipids often react with ROS to generate compounds with altered structures and properties. Oxidative degradation of lipids in biological systems is primarily initiated by iron ion (Fe^n+^) by generating free radicals (O_2_^•−^, NO^•^, ^•^NO_x_, SO_3_^•−^) capable of abstracting protons from PUFAs. The impact of lipid oxidation in cell membranes and how these oxidative events are involved in both physiological processes and major pathological conditions have been analyzed in several reviews (251, 252). Some evidence exists that heme Fe modifies lipid oxidation in muscles (60, 253). At physiological pH and at low levels of PUFAs (<0.5 µM) oxy-Mb does not initiate lipid oxygenation when measured in meat (254). However, in contrast to met-Mb, deoxy-Mb-initiated lipid oxidation is independent of lipid hydroperoxide concentrations (255). In the presence of catalase or superoxide dismutase enzymes, the rate of lipid oxidation is reduced by either met-Mb or oxy-Mb in meat and meat products (62). Does a similar process of lipid oxidation occur in metabolically active biological tissues in situ? The prooxidative activity of deoxy-Mb in biological systems has not been studied in detail, since Mb-initiated lipid oxidation demands strict anaerobic conditions. More experimental evidence is required to evaluate the hypothesis that Mb participates in regulating cellular oxidized lipid pools.

##### 5.3.2.1. summary: mb and lipids.

The results from a diverse biochemistry and molecular modeling literature from multiple laboratories support a role for oxy-Mb as a bona fide LCFA and LCAC binding protein that regulates free and sequestered lipid pools in Mb-expressing tissues. Notably, one study with Mb^−/−^ mice heart showed a threefold higher triacylglyceride (TAG) and diacylglyceride accumulation (190), whereas a different group reported a 25–30% reduction in muscle and heart TAG levels (54). In BAT, larger lipid droplets manifest in Mb^−/−^ mice (19, 20). It is worthy of note that the dissociation constants and binding free energies of LCFAs and LCACs with Mb are comparatively larger (lower affinities) than with other classic lipid binding proteins; i.e., human serum albumin contains 2 high-affinity binding sites, 5 intermediate-affinity binding sites, and 20 low-affinity binding sites of FAs per molecule (256). However, considering the relatively high molar abundance of Mb in type I myofibers and cardiomyocytes (and cold-induced BAT or some cancer cells?), lipid sequestration and trafficking are viable as physiological functions for this protein. These findings support the overarching concept that Mb associates in some way with lipid metabolism and pools in myocytes, cardiomyocytes, and brown adipocytes.

With respect to the hypothesis that lipid binding could alter the signaling roles for Mb (19), recently it was demonstrated by Christen et al. (21) that overexpression in immortalized brown adipocytes of human Mb mutants that do not bind LCFAs led to diminution of several signaling cascades (i.e., cellular O_2_ consumption, PKA phosphorylation in response to forskolin) that had otherwise been induced by overexpression of wild-type human Mb. The latter findings lend credibility to the idea that lipid metabolite binding impacts Mb signaling functions in the cell, but this area is very nascent and requires much more research.

#### 5.3.3. Mb’s possible role in lipid peroxidation.

Unsaturated FAs undergo autooxidation when reacting with molecular O_2_, a process known as lipid peroxidation (66), where both heme and nonheme bound Fe initiate lipid oxidation within muscle-based systems (257, 258). Although MbFe(III) and HbFe(III) have been suggested to play roles in membrane lipid oxidation, the precise catalyst responsible for oxidative deterioration in biological systems remains a topic of considerable debate. Numerous studies examining the prooxidative activity of MbFe(III) and HbFe(III) in simple model systems at physiological pH have failed to confirm any significant catalytic effects (259–262). However, some investigations propose that MbFe(III) exhibits prooxidative behavior only in the presence of peroxides (263, 264). Kanner argued that at physiological pH MbFe(III) needs activation by H_2_O_2_ or lipid hydroperoxides to be an effective prooxidant (265, 266).

The interaction between MbFe(III) and LCFAs at physiological pH results in the reversible formation of the low-spin Fe(III) Mb species hemichrome (260, 267). In contrast, Galaris et al. (61) reported visible absorption spectral changes in MbFe(II) O_2_ upon incubation with linoleic acid at physiological pH, suggesting the formation of the noncatalytic low-spin Mb derivative hemochrome, similar to HbFe(II) O_2_ (268). Most studies indicate that both MbFe(II) O_2_ and MbFe(III) act as prooxidants, and observed differences in their ability to initiate lipid oxidation may be inconsequential (263). This is primarily due to the requirement of strict anaerobic conditions for MbFe(II)-initiated lipid oxidation, to exclude the initiation and subsequent propagation of lipid oxidation by MbFe(II) O_2_. Hogg et al. (264) demonstrated that MbFe(II) O_2_ can promote the oxidative modification of low-density lipoprotein (LDL), a proposition supported by others (269). The presence of reducing agents with the capacity to convert MbFe(IV)=O to MbFe(III) may be a crucial determinant of oxidative damage in biological systems.

#### 5.3.4. Mb-Monocarboxylate interaction and its effect on O_2_ release.

Lactate (LAC) is a three-carbon hydroxycarboxylic acid product of anaerobic metabolism (270). When O_2_ is limiting (anaerobic metabolism), pyruvate (PYR) is reduced to LAC by cytoplasmic LAC dehydrogenase (LDH), oxidizing its cofactor reduced nicotinamide adenine dinucleotide (NADH) in the process (271). Evidence from isolated muscle mitochondria suggests that with the help of monocarboxylic acid transporter (MCT1), a LAC/PYR transporter present on the OMM, LAC can be oxidized because of an intramitochondrial LDH (272–278). Substantial evidence has revealed different roles for LAC, being both a fuel and signaling molecule (270). Earlier studies demonstrated that LAC produced by active muscle is transported to the liver for conversion back to GLU via gluconeogenesis via the Cori cycle (279). However, results have also shown that muscle-derived LAC is transported to the heart as a fuel source for mitochondrial oxidation [intercellular LAC shuttle (270, 279)]. Intracellular LAC formed during glycolysis can be used as an energy source within the same cell (270). The physiological range of tissue LAC spans from 0.5 to 20 mM (280). In exercising skeletal muscles, cellular LAC levels are heightened compared to those in resting muscles, and the LAC-to-PYR ratio reaches ≥80 during intense human exercise (281, 282) This emphasizes that the production of LAC exceeds the rate of oxidative metabolism of PYR under these conditions (283). Elevated LAC levels are associated with a reduction in intracellular pH, dropping from pH 6.8–7.2 to pH 5.0–6.5 (284).

Spectroscopic studies showed that addition of LAC decreases O_2_ affinity (i.e., increase in P_50_) with sperm whale and horse heart oxy-Mb at moderately acidic pH (i.e., pH 6.5, a condition that may be achieved in vivo under a strenuous exercise). However, no spectral changes were detected for the interaction of LAC with deoxy-Mb (12, 285). However, in these studies a LAC binding pocket in Mb crystals was not identified in X-ray diffraction studies even when grown in high concentrations of LAC, implying unresolved stoichiometry. In contrast, our recent studies conducted with ITC have revealed that LAC binds to equine heart Mb in a 1-to-1 stoichiometric ratio with a greater binding affinity with oxy-Mb compared to deoxy-Mb in the range of pH 6.0 to pH 7.0 (LAC did not have binding affinity to deoxy-Mb at pH 7.0) (TABLE 3). A limitation to these interpretations is that ITC measurements are challenging compared to spectroscopic methods because of their unknown protein unfolding, solvation of the ligand, accurate protein-to-ligand ratios, and heats of dilution. In low-affinity ITC studies a 1-to-10 ratio of protein to ligand falls within the permitted limits of *c*-values (i.e., the ratio of concentration of titrate present in the cell to the *K*_d_ value) to determine the accuracy of curve fitting to obtain accurate *K*_d_ and binding stoichiometry values (287). Although there is a lack of precise and straightforward method for determining *K*_d_ value, we believe that the distinct *K*_d_ values displayed in TABLE 3 are due to differences in assay methods. In addition, O_2_ kinetics studies using Clark-type polarographic electrode methods with LAC and oxy-Mb at different pH conditions resulted in rapid O_2_ release in the presence of LAC at a lower pH 6.4 compared to pH 7.0 (286). The complex nature of the pH-dependent changes in O_2_ release from oxy-Mb with the addition of LAC may be attributed to either changes in the protonation states of Mb and/or behaving as a heterotrophic modulator.

**Table 3.** Dissociation constants of Mb interaction with monocarboxylates at different pH conditions

	Sodium Lactate			Sodium Pyruvate		
	pH 7.0	pH 6.4	pH 6.0	pH 7.0	pH 6.4	pH 6.0
Equine heart muscle (data from Refs. 22, 286)						
Oxy-Mb	20.7	6.9	1.9	77.1	218	100.8
Deoxy-Mb		13.7	6.4	0.71	14.2	24.4
Equine heart muscle (data from Ref. 12)						
Oxy-Mb		26,000				
Deoxy-Mb		9,100				
Sperm whale						
Oxy-Mb		26,000				
Deoxy-Mb		2,500				

Dissociation constant (*K*_d_) values (in µM) shown here are calculated from the curve-fitting data in isothermal titration calorimetry (ITC) (286), where *c*-value maintained between 10 and 500 (287), and spectroscopically by tonometric method (12). deoxy-Mb, deoxygenated myoglobin; oxy-Mb, oxygenated myoglobin. Data adapted from Refs. 12, 22, 286.

In the presence of O_2_ (aerobic metabolism), GLU-derived cytosolic PYR enters mitochondria to support tissue energy needs through oxidative phosphorylation, the net result of which is O_2_ consumption and ATP synthesis (279). The physiological concentration of PYR ranges from 0.1 to 0.7 µmol/g in human skeletal muscle and from 30 to 260 µM in blood (288–290). PYR metabolism serves as a branching point for oxidative metabolism, maintenance of tricarboxylic acid (TCA) cycle, resynthesis of GLU (gluconeogenesis), lipid synthesis (de novo lipogenesis), and cholesterol synthesis. For all the anabolic and catabolic pathways, mitochondria play a key role by importing PYR from the cytosol through mitochondrial pyruvate carrier (MPC).

**FIGURE 9.**
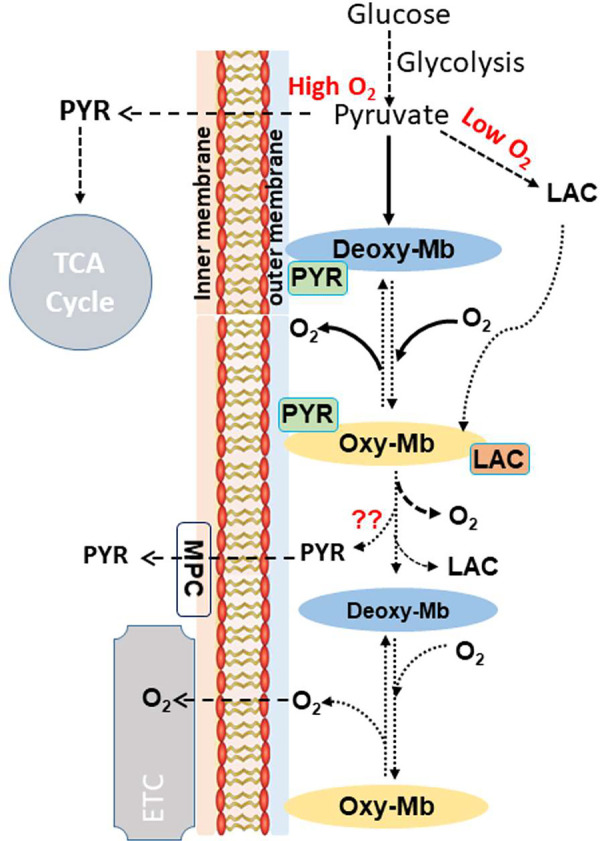
Schematic representation of proposed hypothesis on myoglobin (Mb) interaction with monocarboxylates. The graphical hypothesis displays the possible stoichiometric turnover events on conversion of oxygenated myoglobin (oxy-Mb) to deoxygenated myoglobin (deoxy-Mb) and vice versa in metabolite-bound Mb state in normoxic conditions at a constant supply of diffused oxygen (O_2_) and in hypoxic conditions with reduced Po_2_ and lower pH. Based on isothermal titration calorimetric (ITC) studies, we hypothesize that at high O_2_ aerobic glycolysis generates pyruvate (PYR) that avidly binds to deoxy-Mb (shown by solid arrows) (22), whereas in low-O_2_ conditions lactate (LAC) concentration is increased and may preferentially bind to oxy-Mb (based on ITC studies) and releases O_2_ (based on O_2_ kinetics studies) (286). Solid arrows display high affinity and rapid reaction rates, and dashed arrows display lower affinity and slower reaction rates. ETC, electron transport chain; MPC, mitochondrial pyruvate complex; TCA cycle, tricarboxylic acid cycle; Image adapted from Ref. 22 from *International Journal of Molecular Sciences* per CC-BY 4.0 Open Access.

Similar to results with LAC (286), ITC binding studies have shown that PYR binds to equine heart Mb in a 1-to-1 molar ratio stoichiometry (22). Unlike LAC, PYR binds to both oxy- and deoxy-Mb but with high affinity to the latter at neutral pH. As discussed above, LAC preferentially binds only to oxy-Mb but not to deoxy-Mb at neutral pH, with highest binding affinity of LAC toward oxy-Mb at acidic pH. Conversely, PYR binds deoxy-Mb with more affinity at neutral pH, and its affinity decreases with more acidic conditions (22). Furthermore, in vitro O_2_ release kinetic studies using Clark-type polarographic electrode method with varying concentrations of PYR at neutral (i.e., pH 7.0) and acidic (i.e., pH 6.0–6.5) conditions showed that no release of O_2_ was observed until PYR was added at millimolar concentrations, but such high PYR levels are unlikely inside the cell in vivo. Based on these initial ITC ligand binding results, we hypothesize that in aerobic conditions a transient two-ligand complex (oxy-Mb + PYR+LAC) may be generated only after interaction of O_2_ with the existing deoxy-Mb+PYR complex at neutral pH, since LAC was not found to bind deoxy-Mb at neutral pH (FIGURE 9). Under this model, once the two-ligand complex is formed, PYR may be released instantly, followed by O_2_ and then LAC (22), generating deoxy-Mb in a series of events. Moreover, in intense-exercising muscles or tissues encountering low-O_2_ conditions and elevated cellular LAC levels, a significant reduction in the intracellular pH from pH 6.8–7.2 to pH 5.0–6.5 occurs. Considering the above findings, the hypothetical model where LAC preferential binding to oxy-Mb releases O_2_ and converts Mb into deoxy-Mb is plausible. Thereafter, PYR binding to deoxy-Mb may limit LAC binding, which would tend to promote O_2_ binding and oxy-Mb formation (FIGURE 9). Further investigation on the hypothesized two-ligand binding is required for more insights on structural changes of Mb in ligand bound state(s), and to understand how these dynamics associate with the toggling between oxy- and deoxy-Mb.

##### 5.3.4.1. summary: mb and monocarboxylates.

In biochemical assays, LAC binding to oxy-Mb in acidic conditions rapidly releases O_2_ (286). On the other hand, oxy-Mb associates with the OMM and that contact promotes O_2_ release, converting it to deoxy-Mb. The results of studies examining Mb interactions with LAC and PYR have generated an interesting case that Mb is involved in cellular glycolytic metabolite interaction and trafficking. At neutral pH, PYR has higher affinity to deoxy-Mb, whereas LAC preferentially binds to oxy-Mb. These observations support a working model: as a Mb-expressing cell slips into acidic conditions (i.e., during intense exercise or hypoxic conditions), LAC accumulation leads to LAC-Oxy-Mb binding, which promotes O_2_ release into the cell or at the mitochondrial interface. With this in mind, it is intriguing to consider whether these events modify both LAC and PYR trafficking, glycolysis end product metabolites (LAC and PYR) metabolism, or signaling since PYR binding preferentially occurs with deoxy-Mb. These observations raise another interesting question, “Might the Mb binding dynamics of metabolites from disparate chemical classes influence one another in a way that regulates Mb function?” A case in point is the high-LAC and low-pH condition, which would promote O_2_ off-loading and conversion to deoxy-Mb, which in turn would diminish LCFA and LCAC binding. This could have profound implications for Mb’s cell signaling role and NO homeostasis and for onset and persistence of lipotoxicity under hypoxic conditions.

One other speculative idea is that binding of PYR to deoxy-Mb helps in PYR transport at the mitochondrial membrane, which in turn regulates the metabolic fate of PYR. In a study of renal tissue slices coincubated with Mb and PYR, there was decreased lipid peroxidation and reduced oxidative stress compared to control renal tissue slices (291). Although the mechanism for this was not established, it supports the notion that Mb-PYR interactions can influence cellular physiology. Further comprehensive investigations are needed to address this and other questions related to the functional impacts of LAC and PYR binding to Mb.

## 6. GENE REGULATION OF Mb

### 6.1. Tissue-Specific Mb Gene Expression and Effect of Exercise

Tissue-restricted Mb mRNA expression in various vertebrates is observed in skeletal and cardiac muscles (23, 70, 200, 292). Muscles that are specifically functional for prolonged contractile activity (i.e., cardiac myocytes and “slow-twitch,” mitochondria-rich, oxidative skeletal muscle myofibers) have higher Mb concentrations compared with muscles that are generally adapted for short periods of contractile work (i.e., “fast-twitch,” glycolytic skeletal muscle myofibers). In rabbits, Mb mRNA was induced 15-fold after 21 days of experimental electrical stimulation of the tibialis anterior muscle (25); in contrast, training in humans under normoxic conditions fails to induce Mb mRNA or protein (described in section 3.2 citations). Moreover, in both skeletal muscle and heart, Mb gene expression follows in temporal-spatial pattern with activation of Mb gene expression at specific times during organ development (293). The expression of Mb is induced during brown adipocyte differentiation (19, 21) and during myoblast-to-myotube conversion (11). Mb is not just confined to its expression in skeletal and cardiac muscles or BAT: low-level expression is observed in various nonstriated muscle tissues in some animals (Weddell or gray seals, emperor penguin, fish) and under certain conditions, e.g., hypoxia (292–295). Proteomic studies in mice with exercise training noted an increase in Mb expression in skeletal muscle (296).

### 6.2. Altered Levels of Mb Expression by Diet or Nutrients

Research indicates that the expression levels of Mb vary under different dietary conditions. For instance, the soleus muscle of rats fed with fish oil exhibited a decrease in Mb gene and protein expression compared to rats fed with soybean oil and lard (297). Interestingly, the Mb protein levels in lard-fed rats was also significantly lower than that in soybean oil-fed rats, despite the absence of a significant difference in transcript levels (297). Mb mRNA expression levels in extensor digitorum longus and soleus muscles were also found to be significantly decreased in 48-h food-deprived rats (298). In another study, cultured C2C12 cells (high-glucose DMEM, 5% equine serum, 10 µg/mL insulin, 10 µg/mL transferrin) or 5% lipid-supplemented medium (2 µg/mL arachidonic acid, 10 µg/mL each of linoleic, linolenic, myristic, oleic, palmitic, and stearic fatty acids) revealed that lipid treatments led to higher Mb mRNA expression than control cells in both normoxia and hypoxia conditions via Ca^2+^ signaling pathways involving calcineurin (CN) (52). Western blot assays showed higher Mb protein expression in lipid-treated cells versus control cells, but only in hypoxia conditions (52). Normoxic C2C12 cells were compared with soleus tissue from normoxic rats fed high-fat diets (52). The results revealed higher Mb mRNA expression without a corresponding change in Mb activity (using functional CO-binding assay) under normoxic high-fat conditions compared to C2C12 cells. The results from the studies summarized above suggest that lipids might stimulate Mb expression in skeletal muscles.

### 6.3. Myoglobin “Ectopic” Expression, Splice Variants, and Hypoxia

The “ectopic” (nonmyocyte) occurrence of Mb isoforms was first reported in liver and other organs of hypoxia-tolerant fish models (14, 299). Research studies on human Mb gene structure, transcripts, and promoters by Bicker et al. (300, 301) have identified 16 alternatively spliced variants predominantly expressed in cancer tissue or cell lines in addition to the 3 previously annotated Mb variants. Mb protein in the muscle is encoded by 9 out of the total 19 transcript variants. Among these nine Mb transcripts, muscle-associated Mb transcript variant 2 and alternative protein coding cancer-associated Mb splice variants 9, 10, 11, and 13 are hypoxia inducible. Both standard and alternative cancer-associated Mb variants are expressed not only in cancer tissue and cell lines but also in healthy breast, skeletal, and heart muscle (301, 302). The total sum of alternative Mb variant expression in prostate cancer cells is nearly 96 times higher than the standard Mb variant 2 expression (302). However, the absolute Mb protein concentration in distinct cancer cells is not clear, and there is a need to evaluate how protein levels compare with the highly expressing skeletal and heart muscle tissues.

The evidence that hypoxia and related signals drive Mb expression is somewhat mixed and suggests context-specific regulation. Expression of Mb protein was induced because of longer periods of O_2_ paucity and driven by alternative, tumor-specific hypoxia/hypoxia-inducing factor (HIF)-dependent transcription machinery (295). In another study, cancer-related and hypoxia-responsive Mb mRNA splice variants were specifically expressed in human glioblastoma multiforme (GBM) brain cancer cells under normoxia, where hypoxia-responsive Mb mRNA splice variants in GBM cells were significantly upregulated in hypoxia (303, 304). Further studies have shown that low O_2_ tension regulates Mb protein variant expression in different brain cancers and this expression pattern is associated with a more aggressive phenotype in GBM (303). Additionally, in breast cancer 40% of tissue biopsies are Mb positive, where Mb expression is associated not only with tumor hypoxia markers but also with a positive hormone receptor status and patient prognosis (300). Mb transcripts were upregulated by hypoxia and downregulated by estrogens and androgens in breast and prostate cancer cells, respectively (304). Ectopic Mb is expressed in hormone-independent head and neck squamous cell carcinomas, which is mediated by alternative and hypoxia-inducible transcription (305). In these studies, analysis of the Mb expression on the tissue microarrays revealed significantly lower Mb levels in cancer tissue compared to the normal tissue adjacent to the cancer cells. Loss of Mb expression during differentiation and recovery in induced pluripotent stem cells suggest an undefined functional role of Mb in controlling the balance between aerobic metabolism and pluripotency (306). In human training paradigms under hypoxic conditions, there have been some reports of enhancement of Mb protein or mRNA (173, 176, 179), but this is not a universal finding (176). In mouse C2C12 myocytes, hypoxic culturing conditions significantly increased expression of a Mb reporter construct in a Ca^2+^-dependent manner (295), yet in cultured brown adipocytes hypoxia actually reduced Mb mRNA expression (20).

As noted, Mb is expressed at a low level in some cancers, i.e., human breast and prostate cancer tissues, benign tumors, cancer-adjacent normal tissues, and hyperplastic tissue samples (15–17, 300, 302, 304). However, outside of BAT the biological function and pathophysiological role of “nonmuscle, noncardiac” Mb protein remains unclear. Recent work in mammary epithelial cancer cell lines suggests that induction of Mb expression in some cancers participates in fatty acid trafficking and sequestration (249). In that paper the investigators summarized evidence suggesting a protective effect of Mb expression in carcinomas (perhaps due to reductions in invasiveness and enhanced apoptosis) and contemplated whether these may in part be due to the lipid metabolism role of Mb. In another type of hormone-dependent cancer, increased patient survival rates correlated with higher Mb expression in prostate cancer, which was attributed to tumor-suppressing properties of Mb in cancer cells (300).

The findings described above highlight that Mb is clearly not confined to expression in skeletal muscle, heart, and BAT but also detected in select cancers. Interestingly, the induction in some cancers seems to be induced with hypoxia, indicating that Mb gene regulation is responsive to tissue O_2_ status in some contexts. Might changes in Po_2_ be a trigger for the cold-induced expression in BAT (19, 20), which also displays signs of “hypoxia” due to robust tissue O_2_ consumption during thermogenesis, and hence lowered Po_2_? Does the induction of Mb in cancers point toward a functional role (positive or negative) in cancer initiation or progression?

## 7. CONCLUSIONS AND THE NEXT FRONTIER IN MYOGLOBIN RESEARCH

The conventional model of a physiological function of Mb as an O_2_ storage and carrier protein that supports oxidative phosphorylation is too limited, as the protein is increasingly recognized as a key player in numerous cellular metabolic and signaling pathways. In this review, we aimed to provide a contemporary overview of the key structural and biochemical properties of Mb, coupled to presentation of emerging concepts related to physiological function and Mb gene regulation. Especially considering the multiple tissues and whole body metabolic phenotyping of Mb^−/−^ mice, the weight of evidence does not support the concept that Mb is necessary and sufficient to sustain O_2_ delivery for mitochondrial oxidative phosphorylation and fatty acid oxidation in muscle and heart, at least in this species. Instead, activities of Mb to modulate NO metabolism, bind metabolites, and regulate BAT and cancer cell signaling pathways and metabolism point to other physiological functions. For instance, it has been proposed that Mb has a NO-associated governor role in electron transport chain function. Mb may also be important as a lipid-binding protein that traffics and sequesters LCFAs, LCACs, and some other fats; this may signal a role in prevention of lipotoxicity and in lipid droplet homeostasis/storage of fatty acids.

**FIGURE 10.**
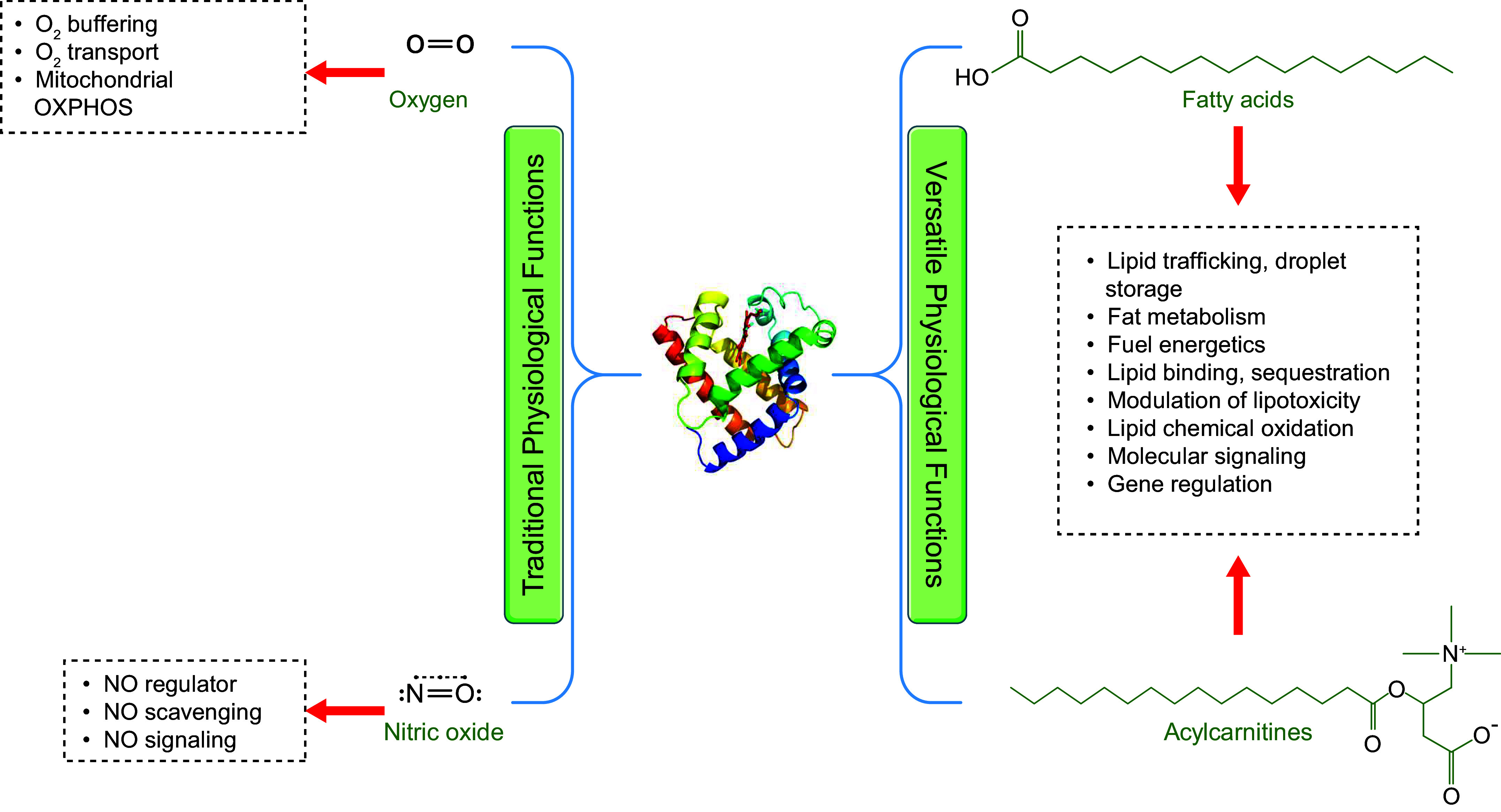
Graphical representation displaying physiological functions of myoglobin (Mb). Mb protein is known to interact with a large group of cellular metabolites (shown in blue brackets) and may concomitantly regulate physiological functions, metabolic cellular pathways, and molecular signaling (shown in dashed boxes with red arrows). NO, nitric oxide; OXPHOS, oxidative phosphorylation.

In addition, we have championed a model in which Mb acts as a Po_2_-sensitive metabolic sensor that modulates downstream signaling cascades and NO/HIF/stress-associated gene and protein regulation. The latter may explain why in some contexts Mb^−/−^ display adaptations such as higher capillary density in muscle and heart, plus altered BAT gene expression patterns, despite no signs of hypoxia in the classic sense. A signaling role for Mb is also consistent with emerging data from cancer models and in brown adipocytes in which Mb abundance has been modified. A critical area for future research should focus on testing this Mb signaling hypothesis and to understand how this is impacted by toggling between deoxy-Mb/met-Mb/oxy-Mb in muscle, heart, BAT, and cancer cells. Another unanswered question is “How might the metabolite milieu and metabolite binding influence Mb’s signaling and other functions?” For instance, one could envision that Mb’s potential function as a metabolic sensor is impacted by changes in tissue workload, blood and tissue oxygenation, and fuel fluxes since these would alter cellular Po_2_, NO, fatty acids, acylcarnitines, pH, lactate, and pyruvate. Under this model of physiological function, Mb plays a central role in metabolic regulation and cell signaling due to rapid conformational changes, gas and metabolite binding properties, and NO modulator functions in response to rapidly changing conditions (FIGURE 10). This perspective aligns well with the observations of G. A. Millikan (3) in his classic 1939 *Physiological Reviews* article “Muscle hemoglobin,” where he referred to the protein as an “intracellular oxygen tension indicator” in light of its rapid transitions between the oxy- and deoxy-forms with a change in tissue O_2_ consumption and Po_2_.

## GRANTS

The body of work on myoglobin by the authors was supported in part through US Department of Agriculture (USDA)-Agricultural Research Service (ARS) project 6026-51000-010-06S, Sturgis Foundation Grant AWD00054750, ACRI-Arkansas Biosciences Institute (ABI) Investigator Initiated Grant Award GR037175-4450S ABI, and the Mouse Metabolic Phenotyping Center consortium [NIH-National Institute of Diabetes and Digestive and Kidney Diseases (NIDDK) U2CDK092993]. S. H. Adams is also funded in part by the Mouse Metabolic Phenotyping Center-*Live* consortium (NIH-NIDDK U2CDK135074).

## DISCLOSURES

S. H. Adams is founder and principal of XenoMed LLC, a company with no competing interests with the studies presented here. None of the other authors has any conflicts of interest, financial or otherwise, to disclose.

## AUTHOR CONTRIBUTIONS

K.K.A., S.H.A., and S.V.C. conceived and designed research; K.K.A., A.A., S.H.A., and S.V.C. prepared figures; K.K.A., A.A., S.H.A., and S.V.C. drafted manuscript; K.K.A., A.A., S.H.A., and S.V.C. edited and revised manuscript; K.K.A., A.A., S.H.A., and S.V.C. approved final version of manuscript.

## References

[1] Möbner KA. Beobachtungen über den Muskelfarbstoff. Nord Med Ark 30: 1–8, 1897. doi:10.1111/j.0954-6820.1897.tb00425.x.

[2] Günther H. Über den Muskelfarbstoff. Virchows Arch Path Anat Physiol Klin Med 230: 146–178, 1921. doi:10.1007/BF01948749.

[3] Millikan GA. Muscle hemoglobin. Physiol Rev 19: 503–523, 1939. doi:10.1152/physrev.1939.19.4.503.

[4] Kendrew JC, Bodo G, Dintzis HM, Parrish RG, Wyckoff H, Phillips DC. A three-dimensional model of the myoglobin molecule obtained by x-ray analysis. Nature 181: 662–666, 1958. doi:10.1038/181662a0. 13517261

[5] Mörner KA. Zur Darstellung and Zusammensetzung der Häminkrystalle. J Intern Med 30: 1–16, 1897. doi:10.1111/j.0954-6820.1897.tb00420.x.

[6] Perutz MF, Rossmann MG, Cullis AF, Muirhead H, Will G, North AC. Structure of hæmoglobin: a three-dimensional Fourier synthesis at 5.5-Å resolution, obtained by x-ray analysis. Nature 185: 416–422, 1960. doi:10.1038/185416a0. 18990801

[7] Wittenberg JB. The molecular mechanism of hemoglobin-facilitated oxygen diffusion. J Biol Chem 241: 104–114, 1966. doi:10.1016/S0021-9258(18)96964-4. 5901041

[8] Moore EG, Gibson QH. Cooperativity in the dissociation of nitric oxide from hemoglobin. J Biol Chem 251: 2788–2794, 1976. doi:10.1016/S0021-9258(17)33557-3. 1262343

[9] Gloster J, Harris P. Fatty acid binding to cytoplasmic proteins of myocardium and red and white skeletal muscle in the rat. A possible new role for myoglobin. Biochem Biophys Res Commun 74: 506–513, 1977. doi:10.1016/0006-291X(77)90333-3. 836303

[10] Doyle MP, Hoekstra JW. Oxidation of nitrogen oxides by bound dioxygen in hemoproteins. J Inorg Biochem 14: 351–358, 1981. doi:10.1016/S0162-0134(00)80291-3. 7276933

[11] Weller PA, Price M, Isenberg H, Edwards YH, Jeffreys AJ. Myoglobin expression: early induction and subsequent modulation of myoglobin and myoglobin mRNA during myogenesis. Mol Cell Biol 6: 4539–4547, 1986. doi:10.1128/mcb.6.12.4539-4547.1986. 3796609 PMC367238

[12] Giardina B, Ascenzi P, Clementi ME, De Sanctis G, Rizzi M, Coletta M. Functional modulation by lactate of myoglobin. A monomeric allosteric hemoprotein. J Biol Chem 271: 16999–17001, 1996. doi:10.1074/jbc.271.29.16999. 8663546

[13] Gödecke A, Flögel U, Zanger K, Ding Z, Hirchenhain J, Decking UK, Schrader J. Disruption of myoglobin in mice induces multiple compensatory mechanisms. Proc Natl Acad Sci USA 96: 10495–10500, 1999. doi:10.1073/pnas.96.18.10495. 10468637 PMC17917

[14] Cossins AR, Williams DR, Foulkes NS, Berenbrink M, Kipar A. Diverse cell-specific expression of myoglobin isoforms in brain, kidney, gill and liver of the hypoxia-tolerant carp and zebrafish. J Exp Biol 212: 627–638, 2009. doi:10.1242/jeb.026286. 19218513

[15] Flonta SE, Arena S, Pisacane A, Michieli P, Bardelli A. Expression and functional regulation of myoglobin in epithelial cancers. Am J Pathol 175: 201–206, 2009. doi:10.2353/ajpath.2009.081124. 19541931 PMC2708806

[16] Kristiansen G, Rose M, Geisler C, Fritzsche FR, Gerhardt J, Lüke C, Ladhoff AM, Knüchel R, Dietel M, Moch H, Varga Z, Theurillat JP, Gorr TA, Dahl E. Endogenous myoglobin in human breast cancer is a hallmark of luminal cancer phenotype. Br J Cancer 102: 1736–1745, 2010. doi:10.1038/sj.bjc.6605702. 20531416 PMC2883703

[17] Gorr TA, Wichmann D, Pilarsky C, Theurillat JP, Fabrizius A, Laufs T, Bauer T, Koslowski M, Horn S, Burmester T, Hankeln T, Kristiansen G. Old proteins—new locations: myoglobin, haemoglobin, neuroglobin and cytoglobin in solid tumours and cancer cells. Acta Physiol (Oxf) 202: 563–581, 2011. doi:10.1111/j.1748-1716.2010.02205.x. 20958924

[18] Chintapalli SV, Jayanthi S, Mallipeddi PL, Gundampati R, Suresh Kumar TK, van Rossum DB, Anishkin A, Adams SH. Novel molecular interactions of acylcarnitines and fatty acids with myoglobin. J Biol Chem 291: 25133–25143, 2016. doi:10.1074/jbc.M116.754978. 27758871 PMC5122780

[19] Blackburn ML, Wankhade UD, Ono-Moore KD, Chintapalli SV, Fox R, Rutkowsky JM, Willis BJ, Tolentino T, Lloyd KC, Adams SH. On the potential role of globins in brown adipose tissue: a novel conceptual model and studies in myoglobin knockout mice. Am J Physiol Endocrinol Metab 321: E47–E62, 2021. doi:10.1152/ajpendo.00662.2020. 33969705 PMC8321818

[20] Aboouf MA, Armbruster J, Thiersch M, Gassmann M, Gödecke A, Gnaiger E, Kristiansen G, Bicker A, Hankeln T, Zhu H, Gorr TA. Myoglobin, expressed in brown adipose tissue of mice, regulates the content and activity of mitochondria and lipid droplets. Biochim Biophys Acta Mol Cell Biol Lipids 1866: 159026, 2021. doi:10.1016/j.bbalip.2021.159026. 34384891

[21] Christen L, Broghammer H, Rapöhn I, Möhlis K, Strehlau C, Ribas-Latre A, Gebhardt C, Roth L, Krause K, Landgraf K, Körner A, Rohde-Zimmermann K, Hoffmann A, Klöting N, Ghosh A, Sun W, Dong H, Wolfrum C, Rassaf T, Hendgen-Cotta UB, Stumvoll M, Blüher M, Heiker JT, Weiner J. Myoglobin-mediated lipid shuttling increases adrenergic activation of brown and white adipocyte metabolism and is as a marker of thermogenic adipocytes in humans. Clin Transl Med 12: e1108, 2022. doi:10.1002/ctm2.1108. 36480426 PMC9731393

[22] Adepu KK, Bhandari D, Anishkin A, Adams SH, Chintapalli SV. Myoglobin-pyruvate interactions: binding thermodynamics, structure-function relationships, and impact on oxygen release kinetics. Int J Mol Sci 23: 8766, 2022. doi:10.3390/ijms23158766. 35955898 PMC9369265

[23] Ordway GA, Garry DJ. Myoglobin: an essential hemoprotein in striated muscle. J Exp Biol 207: 3441–3446, 2004. doi:10.1242/jeb.01172. 15339940

[24] Graber SG, Woodworth RC. Myoglobin expression in L6 muscle cells. Role of differentiation and heme. J Biol Chem 261: 9150–9154, 1986. doi:10.1016/S0021-9258(18)67631-8. 3722191

[25] Underwood LE, Williams RS. Pretranslational regulation of myoglobin gene expression. Am J Physiol Cell Physiol 252: C450–C453, 1987. doi:10.1152/ajpcell.1987.252.4.C450. 3565559

[26] Bekedam MA, van Beek-Harmsen BJ, van Mechelen W, Boonstra A, van der Laarse WJ. Myoglobin concentration in skeletal muscle fibers of chronic heart failure patients. J Appl Physiol (1985) 107: 1138–1143, 2009. doi:10.1152/japplphysiol.00149.2009. 19661455 PMC2763838

[27] van Beek-Harmsen BJ, Bekedam MA, Feenstra HM, Visser FC, van der Laarse WJ. Determination of myoglobin concentration and oxidative capacity in cryostat sections of human and rat skeletal muscle fibres and rat cardiomyocytes. Histochem Cell Biol 121: 335–342, 2004. doi:10.1007/s00418-004-0641-9. 15048578

[28] Sylvén C, Jansson E, Böök K. Myoglobin content in human skeletal muscle and myocardium: relation to fibre size and oxidative capacity. Cardiovasc Res 18: 443–446, 1984. doi:10.1093/cvr/18.7.443. 6744365

[29] Nemeth PM, Lowry OH. Myoglobin levels in individual human skeletal muscle fibers of different types. J Histochem Cytochem 32: 1211–1216, 1984 doi:10.1177/32.11.6491255. 6491255

[30] Masuda K, Truscott K, Lin PC, Kreutzer U, Chung Y, Sriram R, Jue T. Determination of myoglobin concentration in blood-perfused tissue. Eur J Appl Physiol 104: 41–48, 2008. doi:10.1007/s00421-008-0775-x. 18516616

[31] Merx MW, Flögel U, Stumpe T, Gödecke A, Decking UK, Schrader J. Myoglobin facilitates oxygen diffusion. FASEB J 15: 1077–1079, 2001. doi:10.1096/fj.00-0497fje. 11292673

[32] Armstrong RB, Essén-Gustavsson B, Hoppeler H, Jones JH, Kayar SR, Laughlin MH, Lindholm A, Longworth KE, Taylor CR, Weibel ER. O_2_ delivery at VO_2max_ and oxidative capacity in muscles of standardbred horses. J Appl Physiol (1985) 73: 2274–2282, 1992. doi:10.1152/jappl.1992.73.6.2274. 1337073

[33] Olson JS, Phillips GN. Myoglobin discriminates between O_2_, NO, and CO by electrostatic interactions with the bound ligand. J Biol Inorg Chem 2: 544–552, 1997. doi:10.1007/s007750050169.

[34] Noren SR, Williams TM. Body size and skeletal muscle myoglobin of cetaceans: adaptations for maximizing dive duration. Comp Biochem Physiol A Mol Integr Physiol 126: 181–191, 2000. doi:10.1016/S1095-6433(00)00182-3. 10936758

[35] Mirceta S, Signore AV, Burns JM, Cossins AR, Campbell KL, Berenbrink M. Evolution of mammalian diving capacity traced by myoglobin net surface charge. Science 340: 1234192, 2013, doi:10.1126/science.1234192. 23766330

[36] Kooyman GL, Ponganis PJ. The physiological basis of diving to depth: birds and mammals. Annu Rev Physiol 60: 19–32, 1998. doi:10.1146/annurev.physiol.60.1.19. 9558452

[37] Ponganis PJ, Starke LN, Horning M, Kooyman GL. Development of diving capacity in emperor penguins. J Exp Biol 202: 781–786, 1999. doi:10.1242/jeb.202.7.781. 10069967

[38] Arregui M, Singleton EM, Saavedra P, Pabst DA, Moore MJ, Sierra E, Rivero MA, Câmara N, Niemeyer M, Fahlman A, McLellan WA, Bernaldo de Quirós Y. Myoglobin concentration and oxygen stores in different functional muscle groups from three small cetacean species. Animals 11: 451, 2021. doi:10.3390/ani11020451. 33572177 PMC7915992

[39] Scholander PF. Experimental Investigations on the Respiratory Function in Diving Mammals and Birds. Oslo: I kommisjon hos Jacob Dybwad, 1940.

[40] Castellini MA, Somero GN. Buffering capacity of vertebrate muscle: correlations with potentials for anaerobic function. J Comp Physiol B 143: 191–198, 1981. doi:10.1007/BF00797698.

[41] Kooyman GL, Wahrenbrock EA, Castellini MA, Davis RW, Sinnett EE. Aerobic and anaerobic metabolism during voluntary diving in Weddell seals: evidence of preferred pathways from blood chemistry and behavior. J Comp Physiol B 138: 335–346, 1980. doi:10.1007/BF00691568.

[42] Noren SR, Williams TM, Pabst DA, McLellan WA, Dearolf JL. The development of diving in marine endotherms: preparing the skeletal muscles of dolphins, penguins, and seals for activity during submergence. J Comp Physiol B Biochem B 171: 127–134, 2001. doi:10.1007/s003600000161. 11302529

[43] Perkoff GT, Tyler FH. Estimation and physical properties of myoglobin in various species. Metabolism 7: 751–759, 1958. 13600025

[44] Qiu Y, Sutton L, Riggs AF. Identification of myoglobin in human smooth muscle. J Biol Chem 273: 23426–23432, 1998. doi:10.1074/jbc.273.36.23426. 9722578

[45] Cossins A, Berenbrink M. Myoglobin’s new clothes. Nature 454: 416–417, 2008. doi:10.1038/454416a. 18650904

[46] Møller JK, Skibsted LH. Nitric oxide and myoglobins. Chem Rev 102: 1167–1178, 2002. doi:10.1021/cr000078y. 11942791

[47] Eich RF, Li T, Lemon DD, Doherty DH, Curry SR, Aitken JF, Mathews AJ, Johnson KA, Smith RD, Phillips GN, Olson JS. Mechanism of NO-induced oxidation of myoglobin and hemoglobin. Biochemistry 35: 6976–6983, 1996. doi:10.1021/bi960442g. 8679521

[48] Park JW, Piknova B, Dey S, Noguchi CT, Schechter AN. Compensatory mechanisms in myoglobin deficient mice preserve NO homeostasis. Nitric Oxide 90: 10–14, 2019. doi:10.1016/j.niox.2019.06.001. 31173908 PMC7276245

[49] Nakamura M, Nakamura S. Conversion of metmyoglobin to NO myoglobin in the presence of nitrite and reductants. Biochim Biophys Acta 1289: 329–335, 1996. doi:10.1016/0304-4165(95)00161-1. 8620016

[50] Plotnikov EY, Chupyrkina AA, Pevzner IB, Isaev NK, Zorov DB. Myoglobin causes oxidative stress, increase of NO production and dysfunction of kidney’s mitochondria. Biochim Biophys Acta 1792: 796–803, 2009. doi:10.1016/j.bbadis.2009.06.005. 19545623

[51] Kuleva NV, Krasovskaya IE. A New role for myoglobin in cardiac and skeletal muscle function. Biophysics 61: 717–720, 2016. doi:10.1134/S000635091605016X.

[52] Schlater AE, De Miranda MA, Frye MA, Trumble SJ, Kanatous SB. Changing the paradigm for myoglobin: a novel link between lipids and myoglobin. J Appl Physiol (1985) 117: 307–315, 2014. doi:10.1152/japplphysiol.00973.2013. 24925978

[53] Jue T, Shih L, Chung Y. Differential interaction of myoglobin with select fatty acids of carbon chain lengths C8 to C16. Lipids 52: 711–727, 2017. doi:10.1007/s11745-017-4272-z. 28639182

[54] Jue T, Simond G, Wright TJ, Shih L, Chung Y, Sriram R, Kreutzer U, Davis RW. Effect of fatty acid interaction on myoglobin oxygen affinity and triglyceride metabolism. J Physiol Biochem 73: 359–370, 2016. doi:10.1007/s13105-017-0559-z. 28357578

[55] Chintapalli SV, Anishkin A, Adams SH. Exploring the entry route of palmitic acid and palmitoylcarnitine into myoglobin. Arch Biochem Biophys 655: 56–66, 2018. doi:10.1016/j.abb.2018.07.024. 30092229 PMC7413075

[56] Sriram R, Kreutzer U, Shih L, Jue T. Interaction of fatty acid with myoglobin. FEBS Lett 582: 3643–3649, 2008. doi:10.1016/j.febslet.2008.09.047. 18840435 PMC2591068

[57] Chintapalli SV, Bhardwaj G, Patel R, Shah N, Patterson RL, van Rossum DB, Anishkin A, Adams SH. Molecular dynamic simulations reveal the structural determinants of fatty acid binding to oxy-myoglobin. PLoS One 10: e0128496, 2015. doi:10.1371/journal.pone.0128496. 26030763 PMC4451517

[58] Götz FM, Hertel M, Gröschel-Stewart U. Fatty acid binding of myoglobin depends on its oxygenation. Biol Chem Hoppe Seyler 375: 387–392, 1994. doi:10.1515/bchm3.1994.375.6.387. 7980870

[59] Tichivangana JZ, Morrissey PA. Metmyoglobin and inorganic metals as pro-oxidants in raw and cooked muscle systems. Meat Sci 15: 107–116, 1985. doi:10.1016/0309-1740(85)90051-8. 22056129

[60] Rhee KS, Ziprin YA, Ordonez G. Catalysis of lipid oxidation in raw and cooked beef by metmyoglobin-hydrogen peroxide, nonheme iron, and enzyme systems. J Agric Food Chem 35: 1013–1017, 1987. doi:10.1021/jf00078a037.

[61] Galaris D, Sevanian A, Cadenas E, Hochstein P. Ferrylmyoglobin-catalyzed linoleic acid peroxidation. Arch Biochem Biophys 281: 163–169, 1990. doi:10.1016/0003-9861(90)90427-Z. 2383021

[62] Mikkelsen A, Sosniecki L, Skibsted LH. Myoglobin catalysis in lipid oxidation. Eur Food Res Technol 195: 573–573, 1992. doi:10.1007/BF01204568.

[63] Chan WK, Faustman C, Yin M, Decker EA. Lipid oxidation induced by oxymyoglobin and metmyoglobin with involvement of H_2_O_2_ and superoxide anion. Meat Sci 46: 181–190, 1997. doi:10.1016/S0309-1740(97)00014-4. 22062041

[64] Baron CP, Skibsted LH, Andersen HJ. Concentration effects in myoglobin-catalyzed peroxidation of linoleate. J Agric Food Chem 50: 883–888, 2002. doi:10.1021/jf011169e. 11829662

[65] Vuletich JL, Osawa Y, Aviram M. Enhanced lipid oxidation by oxidatively modified myoglobin: role of protein-bound heme. Biochem Biophys Res Commun 269: 647–651, 2000. doi:10.1006/bbrc.2000.2349. 10720470

[66] Baron CP, Andersen HJ. Myoglobin-induced lipid oxidation. a review. J Agric Food Chem 50: 3887–3897, 2002. doi:10.1021/jf011394w. 12083855

[67] Ono-Moore KD, Olfert IM, Rutkowsky JM, Chintapalli SV, Willis BJ, Blackburn ML, Williams DK, O’Reilly J, Tolentino T, Lloyd KC, Adams SH. Metabolic physiology and skeletal muscle phenotypes in male and female myoglobin knockout mice. Am J Physiol Endocrinol Metab 321: E63–E79, 2021. doi:10.1152/ajpendo.00624.2020. 33969704 PMC8321820

[68] Garry DJ, Ordway GA, Lorenz JN, Radford NB, Chin ER, Grange RW, Bassel-Duby R, Williams RS. Mice without myoglobin. Nature 395: 905–908, 1998. doi:10.1038/27681. 9804424

[69] Garry DJ, Meeson A, Yan Z, Williams RS. Life without myoglobin. Cell Mol Life Sci 57: 896–898, 2000. doi:10.1007/PL00000732. 10950305 PMC11146974

[70] Flögel U, Laussmann T, Gödecke A, Abanador N, Schäfers M, Fingas CD, Metzger S, Levkau B, Jacoby C, Schrader J. Lack of myoglobin causes a switch in cardiac substrate selection. Circ Res 96: e68–e75, 2005. doi:10.1161/01.res.0000165481.36288.d2. 15817884

[71] Grange RW, Meeson A, Chin E, Lau KS, Stull JT, Shelton JM, Williams RS, Garry DJ. Functional and molecular adaptations in skeletal muscle of myoglobin-mutant mice. Am J Physiol Cell Physiol 281: C1487–C1494, 2001. doi:10.1152/ajpcell.2001.281.5.C1487. 11600411

[72] Theorell A. Crystalline myoglobin. Biochem Z 252: 1, 1932.

[73] Theorell A. Crystalline myoglobin. VII. Communication. The oxygen equilibrium of myoglobin. Biochem Z 268, 1934.

[74] Theorell A. Crystalline myoglobin. VI. Communication. Myoglobin in equilibrium with oxygen and carbon monoxide. Dissociation constants and heat of combination. Biochem Z 268, 1934.

[75] Theorell A. Crystalline myoglobin. V. Communication. Myoglobin in equilibrium with carbon monoxide. Biochem Z 268, 1934.

[76] Theorell A. Crystalline myoglobin. IV. Communication. Myoglobin in equilibrium with oxygen and carbon monoxide. Biochem Z 268, 1934.

[77] Hill R. Oxygen dissociation curves of muscle haemoglobin. Proc R Soc London B Biol Sci 120: 472–483, 1936. doi:10.1098/rspb.1936.0046.

[78] Hill AV. The combinations of haemoglobin with oxygen and with carbon monoxide. I. Biochem J 7: 471–480, 1913. doi:10.1042/bj0070471. 16742267 PMC1550542

[79] Bonamore A, Boffi A. Flavohemoglobin: structure and reactivity. IUBMB Life 60: 19–28, 2008. doi:10.1002/iub.9. 18379989

[80] Wu G, Wainwright LM, Poole RK. Microbial globins. Adv Microb Physiol 47: 255–310, 2003. doi:10.1016/S0065-2911(03)47005-7. 14560666

[81] Vinogradov SN, Walz DA, Pohajdak B, Moens L, Kapp OH, Suzuki T, Trotman CNA. Adventitious variability? The amino acid sequences of nonvertebrate globins. Comp Biochem Physiol B 106: 1–26, 1993. doi:10.1016/0305-0491(93)90002-M. 8403841

[82] Aliakrinskaia IO. [Tissue hemoglobins in bivalvia (Mollusca)]. Izv Akad Nauk Ser Biol 6: 735–746, 2003. 14994479

[83] Riggs AF. Aspects of the origin and evolution of non-vertebrate hemoglobins. Integr Comp Biol 31: 535–545, 1991. doi:10.1093/icb/31.3.535.

[84] Medeiros R, Serpa L, Brito C, De Wolf H, Jordaens K, Winnepenninckx B, Backeljau T. Radular myoglobin and protein variation within and among some littorinid species (Mollusca: Gastropoda). Hydrobiologia 378: 43–51, 1998. doi:10.1023/A:1003273101855.

[85] Tentori L. Myoglobin, with particular reference to the myoglobin of *Aplysia*. Biochem J 119: 33P–34P, 1970. doi:10.1042/bj1190033P. 5492807 PMC1179501

[86] Bonner AG, Laursen RA. The amino acid sequence of a dimeric myoglobin from the gastropod mollusc. FEBS Lett 73: 201–203, 1977. doi:10.1016/0014-5793(77)80980-0. 838061

[87] Read KR. The radular muscle myoglobin of the gastropod mollusc *Busycon caricum* Gmelin. Comp Biochem Physiol 22: 1–13, 1967. doi:10.1016/0010-406x(67)90161-2. 6049987

[88] Suzuki T, Takao H, Yamanaka K, Gotoh H, Furukohri T, Takagi T. Evidence of met-form myoglobin from *Theliostyla albicilla* radular muscle. Int J Biochem Cell Biol 35: 1119–1126, 2003. doi:10.1016/s1357-2725(03)00034-7. 12672482

[89] Terwilliger RC, Read KR. Oxygen equilibrium studies of the radular muscle myoglobins of the gastropod molluscs, *Buccinum undatum* L. and *Bustcon canaliculatum* L. Int J Biochem 2: 253–261, 1971. doi:10.1016/0020-711X(71)90002-4.

[90] Perutz MF. Regulation of oxygen affinity of hemoglobin: influence of structure of the globin on the heme iron. Annu Rev Biochem 48: 327–386, 1979. doi:10.1146/annurev.bi.48.070179.001551. 382987

[91] Laberge M, Yonetani T. Common dynamics of globin family proteins. IUBMB Life 59: 528–534, 2007. doi:10.1080/15216540701222914. 17701547

[92] Parak FG, Nienhaus GU. Myoglobin, a paradigm in the study of protein dynamics. Chemphyschem 3: 249–254, 2002. doi:10.1002/1439-7641(20020315)3:3<249::AID-CPHC249>3.0.CO;2-A. 12503170

[93] Burmester T, Hankeln T. Function and evolution of vertebrate globins. Acta Physiol (Oxf) 211: 501–514, 2014. doi:10.1111/apha.12312. 24811692

[94] Mathai C, Jourd’heuil FL, Lopez-Soler RI, Jourd’heuil D. Emerging perspectives on cytoglobin, beyond NO dioxygenase and peroxidase. Redox Biol 32: 101468, 2020. doi:10.1016/j.redox.2020.101468. 32087552 PMC7033357

[95] Guidolin D, Tortorella C, Marcoli M, Maura G, Agnati LF. Neuroglobin, a factor playing for nerve cell survival. Int J Mol Sci 17: 1817, 2016. doi:10.3390/ijms17111817. 27809238 PMC5133818

[96] Collman JP, Brauman JI, Doxsee KM. Carbon monoxide binding to iron porphyrins. Proc Natl Acad Sci USA 76: 6035–6039, 1979. doi:10.1073/pnas.76.12.6035. 293699 PMC411794

[97] Schenkman KA, Marble DR, Burns DH, Feigl EO. Myoglobin oxygen dissociation by multiwavelength spectroscopy. J Appl Physiol (1985) 82: 86–92, 1997. doi:10.1152/jappl.1997.82.1.86. 9029202

[98] Olson JS, Mathews AJ, Rohlfs RJ, Springer BA, Egeberg KD, Sligar SG, Tame J, Renaud JP, Nagai K. The role of the distal histidine in myoglobin and haemoglobin. Nature 336: 265–266, 1988. doi:10.1038/336265a0. 3057383

[99] Hlastala MP, McKenna HP, Franada RL, Detter JC. Influence of carbon monoxide on hemoglobin-oxygen binding. J Appl Physiol 41: 893–899, 1976. doi:10.1152/jappl.1976.41.6.893. 12132

[100] Alonso JR, Cardellach F, López S, Casademont J, Miró O. Carbon monoxide specifically inhibits cytochrome C oxidase of human mitochondrial respiratory chain. Pharmacol Toxicol 93: 142–146, 2003. doi:10.1034/j.1600-0773.2003.930306.x. 12969439

[101] Bruce EN, Bruce MC. A multicompartment model of carboxyhemoglobin and carboxymyoglobin responses to inhalation of carbon monoxide. J Appl Physiol (1985) 95: 1235–1247, 2003. doi:10.1152/japplphysiol.00217.2003. 12754170

[102] Coburn RF, Mayers LB. Myoglobin O_2_ tension determined from measurement of carboxymyoglobin in skeletal muscle. Am J Physiol 220: 66–74, 1971. doi:10.1152/ajplegacy.1971.220.1.66. 5538674

[103] Olson KR. Carbon monoxide poisoning: mechanisms, presentation, and controversies in management. J Emerg Med 1: 233–243, 1984. doi:10.1016/0736-4679(84)90078-7. 6491241

[104] Spiro TG, Kozlowski PM. Is the CO adduct of myoglobin bent, and does it matter? Acc Chem Res 34: 137–144, 2001. doi:10.1021/ar000108j. 11263872

[105] Phillips GN, Teodoro ML, Li T, Smith B, Olson JS. Bound CO is a molecular probe of electrostatic potential in the distal pocket of myoglobin. J Phys Chem B 103: 8817–8829, 1999. doi:10.1021/jp9918205.

[106] Olson JS. Kinetic mechanisms for O_2_ binding to myoglobins and hemoglobins. Mol Aspects Med 84: 101024, 2022. doi:10.1016/j.mam.2021.101024. 34544605 PMC8821315

[107] Tian WD, Sage JT, Champion PM. Investigations of ligand association and dissociation rates in the “open” and “closed” states of myoglobin. J Mol Biol 233: 155–166, 1993. doi:10.1006/jmbi.1993.1491. 8377182

[108] Yang F, Phillips GN. Crystal structures of CO-, deoxy- and met-myoglobins at various pH values. J Mol Biol 256: 762–774, 1996. doi:10.1006/jmbi.1996.0123. 8642596

[109] Salter MD, Blouin GC, Soman J, Singleton EW, Dewilde S, Moens L, Pesce A, Nardini M, Bolognesi M, Olson JS. Determination of ligand pathways in globins: apolar tunnels versus polar gates. J Biol Chem 287: 33163–33178, 2012. doi:10.1074/jbc.M112.392258. 22859299 PMC3460423

[110] Boechi L, Arrar M, Martí MA, Olson JS, Roitberg AE, Estrin DA. Hydrophobic effect drives oxygen uptake in myoglobin via histidine E7. J Biol Chem 288: 6754–6762, 2013. doi:10.1074/jbc.M112.426056. 23297402 PMC3585112

[111] Scott EE, Gibson QH, Olson JS. Mapping the pathways for O_2_ entry into and exit from myoglobin. J Biol Chem 276: 5177–5188, 2001. doi:10.1074/jbc.M008282200. 11018046

[112] Olson JS, Phillips GN. Kinetic pathways and barriers for ligand binding to myoglobin. J Biol Chem 271: 17593–17596, 1996. doi:10.1074/jbc.271.30.17593. 8698688

[113] Vanek T, Kohli A. Biochemistry, myoglobin. In: *StatPearls*. Treasure Island, FL: StatPearls Publishing, Inc., 2023. 31334976

[114] Jürgens KD, Papadopoulos S, Peters T, Gros G. Myoglobin: just an oxygen store or also an oxygen transporter? News Physiol Sci 15: 269–274, 2000. doi:10.1152/physiologyonline.2000.15.5.269. 11390925

[115] Takakura H, Furuichi Y, Yamada T, Jue T, Ojino M, Hashimoto T, Iwase S, Hojo T, Izawa T, Masuda K. Endurance training facilitates myoglobin desaturation during muscle contraction in rat skeletal muscle. Sci Rep 5: 9403–9407, 2015. doi:10.1038/srep09403. 25801957 PMC4371155

[116] Okushima D, Poole DC, Rossiter HB, Barstow TJ, Kondo N, Ohmae E, Koga S. Muscle deoxygenation in the quadriceps during ramp incremental cycling: deep vs. superficial heterogeneity. J Appl Physiol (1985) 119: 1313–1319, 2015. doi:10.1152/japplphysiol.00574.2015. 26404619

[117] Takagi S, Murase N, Kime R, Niwayama M, Osada T, Katsumura T. Skeletal muscle deoxygenation abnormalities in early post-myocardial infarction. Med Sci Sports Exerc 46: 2062–2069, 2014. doi:10.1249/MSS.0000000000000334. 24621961

[118] Ienaga K, Yamaguchi K, Ota N, Goto K. Augmented muscle deoxygenation during repeated sprint exercise with post-exercise blood flow restriction. Physiol Rep 10: e15294, 2022. doi:10.14814/phy2.15294. 35586958 PMC9117971

[119] Antunes A, Domingos C, Diniz L, Monteiro CP, Espada MC, Alves FB, Reis JF. The relationship between VO_2_ and muscle deoxygenation kinetics and upper body repeated sprint performance in trained judokas and healthy individuals. Int J Environ Res Public Health 19: 861, 2022. doi:10.3390/ijerph19020861. 35055684 PMC8776052

[120] Richardson RS, Leigh JS, Wagner PD, Noyszewski EA. Cellular PO_2_ as a determinant of maximal mitochondrial O_2_ consumption in trained human skeletal muscle. J Appl Physiol (1985) 87: 325–331, 1999. doi:10.1152/jappl.1999.87.1.325. 10409591

[121] Chung Y, Molé PA, Sailasuta N, Tran TK, Hurd R, Jue T. Control of respiration and bioenergetics during muscle contraction. Am J Physiol Cell Physiol 288: C730–C738, 2005. doi:10.1152/ajpcell.00138.2004. 15537712

[122] Molé PA, Chung Y, Tran TK, Sailasuta N, Hurd R, Jue T. Myoglobin desaturation with exercise intensity in human gastrocnemius muscle. Am J Physiol Regul Integr Comp Physiol 277: R173–R180, 1999. doi:10.1152/ajpregu.1999.277.1.R173. 10409271

[123] Ponganis PJ, Kreutzer U, Stockard TK, Lin PC, Sailasuta N, Tran TK, Hurd R, Jue T. Blood flow and metabolic regulation in seal muscle during apnea. J Exp Biol 211: 3323–3332, 2008. doi:10.1242/jeb.018887. 18840667

[124] Richardson RS, Noyszewski EA, Kendrick KF, Leigh JS, Wagner PD. Myoglobin O_2_ desaturation during exercise. Evidence of limited O_2_ transport. J Clin Invest 96: 1916–1926, 1995. doi:10.1172/JCI118237. 7560083 PMC185828

[125] Sugawara Y, Shikama K. Autoxidation of native oxymyoglobin. Thermodynamic analysis of the pH profile. Eur J Biochem 110: 241–246, 1980. doi:10.1111/j.1432-1033.1980.tb04861.x. 6254762

[126] Brantley RE, Smerdon SJ, Wilkinson AJ, Singleton EW, Olson JS. The mechanism of autooxidation of myoglobin. J Biol Chem 268: 6995–7010, 1993. doi:10.1016/S0021-9258(18)53138-0. 8463233

[127] Wright TJ, Davis RW. Myoglobin extraction from mammalian skeletal muscle and oxygen affinity determination under physiological conditions. Protein Expr Purif 107: 50–55, 2015. doi:10.1016/j.pep.2014.11.004. 25462805

[128] Arcon JP, Rosi P, Petruk AA, Marti MA, Estrin DA. Molecular mechanism of myoglobin autoxidation: insights from computer simulations. J Phys Chem B 119: 1802–1813, 2015. doi:10.1021/jp5093948. 25578484

[129] Gutzke D, Trout GR. Temperature and pH dependence of the autoxidation rate of bovine, ovine, porcine, and cervine oxymyoglobin isolated from three different muscles—longissimus dorsi, gluteus medius, and biceps femoris. J Agric Food Chem 50: 2673–2678, 2002. doi:10.1021/jf0112769. 11958640

[130] Draghi F, Miele AE, Travaglini-Allocatelli C, Vallone B, Brunori M, Gibson QH, Olson JS. Controlling ligand binding in myoglobin by mutagenesis. J Biol Chem 277: 7509–7519, 2002. doi:10.1074/jbc.M109206200. 11744723

[131] Carver TE, Brantley RE, Singleton EW, Arduini RM, Quillin ML, Phillips GN, Olson JS. A novel site-directed mutant of myoglobin with an unusually high O_2_ affinity and low autooxidation rate. J Biol Chem 267: 14443–14450, 1992. doi:10.1016/S0021-9258(19)49732-9. 1629229

[132] Wittenberg BA, Wittenberg JB. Myoglobin-mediated oxygen delivery to mitochondria of isolated cardiac myocytes. Proc Natl Acad Sci USA 84: 7503–7507, 1987. doi:10.1073/pnas.84.21.7503. 3118370 PMC299324

[133] Yamada T, Furuichi Y, Takakura H, Hashimoto T, Hanai Y, Jue T, Masuda K. Interaction between myoglobin and mitochondria in rat skeletal muscle. J Appl Physiol (1985) 114: 490–497, 2013. doi:10.1152/japplphysiol.00789.2012. 23195625

[134] Basova LV, Tiktopulo EI, Bychkova VE. Model phospholipid membranes affect the tertiary structure of holomyoglobin: conformational changes at pH 6.2. Mol Biol 39: 105–112, 2005. doi:10.1007/s11008-005-0015-y. 15773556

[135] Svedenhag J, Henriksson J, Sylvén C. Dissociation of training effects on skeletal muscle mitochondrial enzymes and myoglobin in man. Acta Physiol Scand 117: 213–218, 1983. doi:10.1111/j.1748-1716.1983.tb07199.x. 6306998

[136] Masuda K, Okazaki K, Kuno S, Asano K, Shimojo H, Katsuta S. Endurance training under 2500-m hypoxia does not increase myoglobin content in human skeletal muscle. Eur J Appl Physiol 85: 486–490, 2001. doi:10.1007/s004210100471. 11606019

[137] Burwell LS, Nadtochiy SM, Tompkins AJ, Young S, Brookes PS. Direct evidence for S-nitrosation of mitochondrial complex I. Biochem J 394: 627–634, 2006. doi:10.1042/BJ20051435. 16371007 PMC1383712

[138] Postnikova GB, Shekhovtsova EA. Fluorescence studies on the interaction of myoglobin with mitochondria. Biochemistry (Mosc) 77: 280–287, 2012. doi:10.1134/S0006297912030066. 22803945

[139] Postnikova GB, Tselikova SV. [Myoglobin and mitochondria: kinetics of oxymyoglobin deoxygenation in mitochondria suspension]. Biofizika 50: 297–306, 2005. 15856988

[140] Postnikova GB, Shekhovtsova EA. Myoglobin and mitochondria: how does the “oxygen store” work? J Phys Chem Biophys 3: 4, 2013. doi:10.4172/2161-0398.1000126.

[141] Anishkin A, Adepu KK, Bhandari D, Adams SH, Chintapalli SV. Computational analysis reveals unique binding patterns of oxygenated and deoxygenated myoglobin to the outer mitochondrial membrane. Biomolecules 13: 1138, 2023. doi:10.3390/biom13071138. 37509174 PMC10377724

[142] Nelson BD, Fleischer S. Phospholipid requirements for the reconstitution of complex-III vesicles exhibiting controlled electron transport. Biochem J 194: 783–787, 1981. doi:10.1042/bj1940783. 6272738 PMC1162813

[143] Sun F, Huo X, Zhai Y, Wang A, Xu J, Su D, Bartlam M, Rao Z. Crystal structure of mitochondrial respiratory membrane protein complex II. Cell 121: 1043–1057, 2005. doi:10.1016/j.cell.2005.05.025. 15989954

[144] Shinzawa-Itoh K, Aoyama H, Muramoto K, Terada H, Kurauchi T, Tadehara Y, Yamasaki A, Sugimura T, Kurono S, Tsujimoto K, Mizushima T, Yamashita E, Tsukihara T, Yoshikawa S. Structures and physiological roles of 13 integral lipids of bovine heart cytochrome c oxidase. EMBO J 26: 1713–1725, 2007. doi:10.1038/sj.emboj.7601618. 17332748 PMC1829383

[145] Sharpley MS, Shannon RJ, Draghi F, Hirst J. Interactions between phospholipids and NADH:ubiquinone oxidoreductase (complex I) from bovine mitochondria. Biochemistry 45: 241–248, 2006. doi:10.1021/bi051809x. 16388600

[146] Koma R, Shibaguchi T, Pérez López C, Oka T, Jue T, Takakura H, Masuda K. Localization of myoglobin in mitochondria: implication in regulation of mitochondrial respiration in rat skeletal muscle. Physiol Rep 9: e14769, 2021. doi:10.14814/phy2.14769. 33650803 PMC7923563

[147] Postnikova GB, Tselikova SV, Shekhovtsova EA. Myoglobin and mitochondria: oxymyoglobin interacts with mitochondrial membrane during deoxygenation. Biochemistry (Mosc) 74: 1211–1218, 2009. doi:10.1134/S0006297909110054. 19916935

[148] Postnikova GB, Shekhovtsova EA. Effect of artificial and natural phospholipid membranes on rate of sperm whale oxymyoglobin autooxidation. Biochemistry (Mosc) 78: 267–272, 2013. doi:10.1134/S0006297913030085. 23586720

[149] Postnikova GB, Shekhovtsova EA. Myoglobin: oxygen depot or oxygen transporter to mitochondria? A novel mechanism of myoglobin deoxygenation in cells. Biochemistry (Mosc) 83: 168–183, 2018. doi:10.1134/S0006297918020098. 29618303

[150] Wittenberg BA, Wittenberg JB, Caldwell PR. Role of myoglobin in the oxygen supply to red skeletal muscle. J Biol Chem 250: 9038–9043, 1975. doi:10.1016/S0021-9258(19)40690-X. 1194276

[151] Nadege B, Lestienne P, Rossignol R. Mitochondria: from bioenergetics to the metabolic regulation of carcinogenesis. Front Biosci 14: 4015–4034, 2009. doi:10.2741/3509. 19273331

[152] Baracca A, Chiaradonna F, Sgarbi G, Solaini G, Alberghina L, Lenaz G. Mitochondrial Complex I decrease is responsible for bioenergetic dysfunction in K-ras transformed cells. Biochim Biophys Acta 1797: 314–323, 2010. doi:10.1016/j.bbabio.2009.11.006. 19931505

[153] Porporato PE, Filigheddu N, Pedro JM, Kroemer G, Galluzzi L. Mitochondrial metabolism and cancer. Cell Res 28: 265–280, 2018. doi:10.1038/cr.2017.155. 29219147 PMC5835768

[154] Bertout JA, Patel SA, Simon MC. The impact of O_2_ availability on human cancer. Nat Rev Cancer 8: 967–975, 2008. doi:10.1038/nrc2540. 18987634 PMC3140692

[155] Denko NC. Hypoxia, HIF1 and glucose metabolism in the solid tumour. Nat Rev Cancer 8: 705–713, 2008. doi:10.1038/nrc2468. 19143055

[156] Nizet V, Johnson RS. Interdependence of hypoxic and innate immune responses. Nat Rev Immunol 9: 609–617, 2009. doi:10.1038/nri2607. 19704417 PMC4343208

[157] Solaini G, Harris DA. Biochemical dysfunction in heart mitochondria exposed to ischaemia and reperfusion. Biochem J 390: 377–394, 2005. doi:10.1042/BJ20042006. 16108756 PMC1198918

[158] Liu B, Tewari AK, Zhang L, Green-Church KB, Zweier JL, Chen YR, He G. Proteomic analysis of protein tyrosine nitration after ischemia reperfusion injury: mitochondria as the major target. Biochim Biophys Acta 1794: 476–485, 2009. doi:10.1016/j.bbapap.2008.12.008. 19150419 PMC2637933

[159] Bosetti F, Brizzi F, Barogi S, Mancuso M, Siciliano G, Tendi EA, Murri L, Rapoport SI, Solaini G. Cytochrome c oxidase and mitochondrial F1F0-ATPase (ATP synthase) activities in platelets and brain from patients with Alzheimer’s disease. Neurobiol Aging 23: 371–376, 2002. doi:10.1016/S0197-4580(01)00314-1. 11959398

[160] Peers C, Pearson HA, Boyle JP. Hypoxia and Alzheimer’s disease. Essays Biochem 43: 153–164, 2007. doi:10.1042/bse0430153. 17705799

[161] Catrina SB, Okamoto K, Pereira T, Brismar K, Poellinger L. Hyperglycemia regulates hypoxia-inducible factor-1α protein stability and function. Diabetes 53: 3226–3232, 2004. doi:10.2337/diabetes.53.12.3226. 15561954

[162] Sack MN. Type 2 diabetes, mitochondrial biology and the heart. J Mol Cell Cardiol 46: 842–849, 2009. doi:10.1016/j.yjmcc.2009.02.001. 19217910 PMC2683201

[163] Jezek P, Plecitá-Hlavatá L. Mitochondrial reticulum network dynamics in relation to oxidative stress, redox regulation, and hypoxia. Int J Biochem Cell Biol 41: 1790–1804, 2009. doi:10.1016/j.biocel.2009.02.014. 19703650

[164] Spinazzi M, Cazzola S, Bortolozzi M, Baracca A, Loro E, Casarin A, Solaini G, Sgarbi G, Casalena G, Cenacchi G, Malena A, Frezza C, Carrara F, Angelini C, Scorrano L, Salviati L, Vergani L. A novel deletion in the GTPase domain of OPA1 causes defects in mitochondrial morphology and distribution, but not in function. Hum Mol Genet 17: 3291–3302, 2008. doi:10.1093/hmg/ddn225. 18678599

[165] Parone PA, Da Cruz S, Tondera D, Mattenberger Y, James DI, Maechler P, Barja F, Martinou JC. Preventing mitochondrial fission impairs mitochondrial function and leads to loss of mitochondrial DNA. PLoS One 3: e3257, 2008. doi:10.1371/journal.pone.0003257. 18806874 PMC2532749

[166] Chen H, Chomyn A, Chan DC. Disruption of fusion results in mitochondrial heterogeneity and dysfunction. J Biol Chem 280: 26185–26192, 2005. doi:10.1074/jbc.M503062200. 15899901

[167] Bakker A, Goossens F, De Bie M, Bernaert I, Van Belle H, Jacob W. The effect of ischemia and reperfusion on mitochondrial contact sites in isolated rat hearts. Histol Histopathol 10: 405–416, 1995. 7599437

[168] Yamada T, Takakura H, Jue T, Hashimoto T, Ishizawa R, Furuichi Y, Kato Y, Iwanaka N, Masuda K. Myoglobin and the regulation of mitochondrial respiratory chain complex IV. J Physiol 594: 483–495, 2016. doi:10.1113/JP270824. 26584944 PMC4713734

[169] Salathe EP, Chen C. The role of myoglobin in retarding oxygen depletion in skeletal muscle. Math Biosci 116: 1–20, 1993. doi:10.1016/0025-5564(93)90059-J. 8343616

[170] Conley KE, Jones C. Myoglobin content and oxygen diffusion: model analysis of horse and steer muscle. Am J Physiol Cell Physiol 271: C2027–C2036, 1996. doi:10.1152/ajpcell.1996.271.6.C2027. 8997205

[171] Jürgens KD, Peters T, Gros G. Diffusivity of myoglobin in intact skeletal muscle cells. Proc Natl Acad Sci USA 91: 3829–3833, 1994. doi:10.1073/pnas.91.9.3829. 8170996 PMC43675

[172] Papadopoulos S, Endeward V, Revesz-Walker B, Jurgens KD, Gros G. Radial and longitudinal diffusion of myoglobin in single living heart and skeletal muscle cells. Proc Natl Acad Sci U S A 98: 5904–5909, 2001. doi:10.1073/pnas.101109798. 11320218 PMC33311

[173] Terrados N, Jansson E, Sylvén C, Kaijser L. Is hypoxia a stimulus for synthesis of oxidative enzymes and myoglobin? J Appl Physiol (1985) 68: 2369–2372, 1990. doi:10.1152/jappl.1990.68.6.2369. 2384418

[174] Terrados N, Melichna J, Sylvén C, Jansson E. Decrease in skeletal muscle myoglobin with intensive training in man. Acta Physiol Scand 128: 651–652, 1986. doi:10.1111/j.1748-1716.1986.tb08026.x. 3811991

[175] Möller P, Brandt R. The effect of physical training in elderly subjects with special reference to energy-rich phosphagens and myoglobin in leg skeletal muscle. Clin Physiol 2: 307–314, 1982. doi:10.1111/j.1475-097X.1982.tb00035.x. 6751657

[176] Brocherie F, Millet GP, D’Hulst G, Van Thienen R, Deldicque L, Girard O. Repeated maximal-intensity hypoxic exercise superimposed to hypoxic residence boosts skeletal muscle transcriptional responses in elite team-sport athletes. Acta Physiol (Oxf) 222: e12851, 2018. doi:10.1111/apha.12851. 28103427

[177] Friedmann B, Kinscherf R, Borisch S, Richter G, Bärtsch P, Billeter R. Effects of low-resistance/high-repetition strength training in hypoxia on muscle structure and gene expression. Pflugers Arch 446: 742–751, 2003. doi:10.1007/s00424-003-1133-9. 12861415

[178] Ookawara T, Suzuk K, Haga S, Ha S, Chung KS, Toshinai K, Hamaoka T, Katsumura T, Takemasa T, Mizuno M, Hitomi Y, Kizaki T, Suzuki K, Ohno H. Transcription regulation of gene expression in human skeletal muscle in response to endurance training. Res Commun Mol Pathol Pharmacol 111: 41–54, 2002. 14632313

[179] Vogt M, Puntschart A, Geiser J, Zuleger C, Billeter R, Hoppeler H. Molecular adaptations in human skeletal muscle to endurance training under simulated hypoxic conditions. J Appl Physiol (1985) 91: 173–182, 2001. doi:10.1152/jappl.2001.91.1.173. 11408428

[180] Jansson E, Sylvén C, Nordevang E. Myoglobin in the quadriceps femoris muscle of competitive cyclists and untrained men. Acta Physiol Scand 114: 627–629, 1982. doi:10.1111/j.1748-1716.1982.tb07035.x. 7136792

[181] Terrados N. Altitude training and muscular metabolism. Int J Sports Med 13, Suppl 1: S206–S209, 1992. doi:10.1055/s-2007-1024641. 1483777

[182] Neufer PD, Ordway GA, Williams RS. Transient regulation of c-fos, αB-crystallin, and hsp70 in muscle during recovery from contractile activity. Am J Physiol Cell Physiol 274: C341–C346, 1998. doi:10.1152/ajpcell.1998.274.2.C341. 9486122

[183] Meeson AP, Radford N, Shelton JM, Mammen PP, DiMaio JM, Hutcheson K, Kong Y, Elterman J, Williams RS, Garry DJ. Adaptive mechanisms that preserve cardiac function in mice without myoglobin. Circ Res 88: 713–720, 2001. doi:10.1161/hh0701.089753. 11304494

[184] Endeward V, Gros G, Jürgens KD. Significance of myoglobin as an oxygen store and oxygen transporter in the intermittently perfused human heart: a model study. Cardiovasc Res 87: 22–29, 2010. doi:10.1093/cvr/cvq036. 20124401

[185] Norvell JC, Nunes AC, Schoenborn BP. Neutron diffraction analysis of myoglobin: structure of the carbon monoxide derivative. Science 190: 568–570, 1975. doi:10.1126/science.1188354. 1188354

[186] Schlieper G, Kim JH, Molojavyi A, Jacoby C, Laussmann T, Flögel U, Gödecke A, Schrader J. Adaptation of the myoglobin knockout mouse to hypoxic stress. Am J Physiol Regul Integr Comp Physiol 286: R786–R792, 2004. doi:10.1152/ajpregu.00043.2003. 14656764

[187] Gödecke A, Molojavyi A, Heger J, Flögel U, Ding Z, Jacoby C, Schrader J. Myoglobin protects the heart from inducible nitric-oxide synthase (iNOS)-mediated nitrosative stress. J Biol Chem 278: 21761–21766, 2003. doi:10.1074/jbc.M302573200. 12665503

[188] Molojavyi A, Lindecke A, Raupach A, Moellendorf S, Köhrer K, Gödecke A. Myoglobin-deficient mice activate a distinct cardiac gene expression program in response to isoproterenol-induced hypertrophy. Physiol Genomics 41: 137–145, 2010. doi:10.1152/physiolgenomics.90297.2008. 20145201

[189] Flögel U, Gödecke A, Klotz LO, Schrader J. Role of myoglobin in the antioxidant defense of the heart. FASEB J 18: 1156–1158, 2004. doi:10.1096/fj.03-1382fje. 15132981

[190] Hendgen-Cotta UB, Esfeld S, Coman C, Ahrends R, Klein-Hitpass L, Flögel U, Rassaf T, Totzeck M. A novel physiological role for cardiac myoglobin in lipid metabolism. Sci Rep 7: 43219, 2017. doi:10.1038/srep43219. 28230173 PMC5322402

[191] Merx MW, Gödecke A, Flögel U, Schrader J. Oxygen supply and nitric oxide scavenging by myoglobin contribute to exercise endurance and cardiac function. FASEB J 19: 1015–1017, 2005. doi:10.1096/fj.04-2886fje. 15817640

[192] Zoladz JA, Grandys M, Smeda M, Kij A, Kurpinska A, Kwiatkowski G, Karasinski J, Hendgen-Cotta U, Chlopicki S, Majerczak J. Myoglobin deficiency impairs maximal oxygen uptake and exercise performance: a lesson from Mb^−/−^ mice. J Physiol 602: 855–873, 2024. doi:10.1113/JP285067. 38376957

[193] O’Brien KM, Sidell BD. The interplay among cardiac ultrastructure, metabolism and the expression of oxygen-binding proteins in Antarctic fishes. J Exp Biol 203: 1287–1297, 2000. doi:10.1242/jeb.203.8.1287. 10729278

[194] Macqueen DJ, Garcia de la Serrana DG, Johnston IA. Cardiac myoglobin deficit has evolved repeatedly in teleost fishes. Biol Lett 10: 20140225, 2014. doi:10.1098/rsbl.2014.0225. 24919701 PMC4090546

[195] Dalziel AC, Schulte PM. Correlates of prolonged swimming performance in F2 hybrids of migratory and non-migratory threespine stickleback. J Exp Biol 215: 3587–3596, 2012. doi:10.1242/jeb.071951. 22771745

[196] Butler AR, Williams DL. The physiological role of nitric oxide. Chem Soc Rev 22: 233, 1993. doi:10.1039/cs9932200233.

[197] Nisoli E, Carruba MO. Nitric oxide and mitochondrial biogenesis. J Cell Sci 119: 2855–2862, 2006. doi:10.1242/jcs.03062. 16825426

[198] Erusalimsky JD, Moncada S. Nitric oxide and mitochondrial signaling: from physiology to pathophysiology. Arterioscler Thromb Vasc Biol 27: 2524–2531, 2007. doi:10.1161/ATVBAHA.107.151167. 17885213

[199] Wylie LJ, Park JW, Vanhatalo A, Kadach S, Black MI, Stoyanov Z, Schechter AN, Jones AM, Piknova B. Human skeletal muscle nitrate store: influence of dietary nitrate supplementation and exercise. J Physiol 597: 5565–5576, 2019. doi:10.1113/JP278076. 31350908 PMC9358602

[200] Flögel U, Merx MW, Godecke A, Decking UK, Schrader J. Myoglobin: a scavenger of bioactive NO. Proc Natl Acad Sci USA 98: 735–740, 2001. doi:10.1073/pnas.98.2.735. 11136228 PMC14657

[201] Brookes PS, Levonen AL, Shiva S, Sarti P, Darley-Usmar VM. Mitochondria: regulators of signal transduction by reactive oxygen and nitrogen species. Free Radic Biol Med 33: 755–764, 2002. doi:10.1016/S0891-5849(02)00901-2. 12208364

[202] Poderoso JJ, Carreras MC, Lisdero C, Riobó N, Schöpfer F, Boveris A. Nitric oxide inhibits electron transfer and increases superoxide radical production in rat heart mitochondria and submitochondrial particles. Arch Biochem Biophys 328: 85–92, 1996. doi:10.1006/abbi.1996.0146. 8638942

[203] Levine AB, Punihaole D, Levine TB. Characterization of the role of nitric oxide and its clinical applications. Cardiology 122: 55–68, 2012. doi:10.1159/000338150. 22722323

[204] Moncada S, Erusalimsky JD. Does nitric oxide modulate mitochondrial energy generation and apoptosis? Nat Rev Mol Cell Biol 3: 214–220, 2002. doi:10.1038/nrm762. 11994742

[205] Walters CL, Taylor AM. Pig-heart myoglobin and its derivatives with nitric oxide. Biochim Biophys Acta 82: 420–422, 1964. doi:10.1016/0304-4165(64)90320-4. 14127975

[206] Clanton TL. Managing the power grid: how myoglobin can regulate PO_2_ and energy distribution in skeletal muscle. J Appl Physiol (1985) 126: 787–790, 2019. doi:10.1152/japplphysiol.00614.2018. 30335576

[207] Koebke KJ, Waletzko MT, Pacheco AA. Direct monitoring of the reaction between photochemically generated nitric oxide and *Mycobacterium tuberculosis* truncated hemoglobin N wild type and variant forms: an assessment of computational mechanistic predictions. Biochemistry 55: 686–696, 2016. doi:10.1021/acs.biochem.5b01145. 26757411

[208] Foley EL, Hvitved AN, Eich RF, Olson JS. Mechanisms of nitric oxide reactions with globins using mammalian myoglobin as a model system. J Inorg Biochem 233: 111839, 2022. doi:10.1016/j.jinorgbio.2022.111839. 35599166

[209] Smith RD, Blouin GC, Johnson KA, Phillips GN, Olson JS. Straight-chain alkyl isocyanides open the distal histidine gate in crystal structures of myoglobin. Biochemistry 49: 4977–4986, 2010. doi:10.1021/bi1001739. 20481504 PMC4074459

[210] Quillin ML, Arduini RM, Olson JS, Phillips GN. High-resolution crystal structures of distal histidine mutants of sperm whale myoglobin. J Mol Biol 234: 140–155, 1993. doi:10.1006/jmbi.1993.1569. 8230194

[211] Shiva S, Huang Z, Grubina R, Sun J, Ringwood LA, MacArthur PH, Xu X, Murphy E, Darley-Usmar VM, Gladwin MT. Deoxymyoglobin is a nitrite reductase that generates nitric oxide and regulates mitochondrial respiration. Circ Res 100: 654–661, 2007. doi:10.1161/01.RES.0000260171.52224.6b. 17293481

[212] Rassaf T, Flögel U, Drexhage C, Hendgen-Cotta U, Kelm M, Schrader J. Nitrite reductase function of deoxymyoglobin: oxygen sensor and regulator of cardiac energetics and function. Circ Res 100: 1749–1754, 2007. doi:10.1161/CIRCRESAHA.107.152488. 17495223

[213] Hendgen-Cotta UB, Merx MW, Shiva S, Schmitz J, Becher S, Klare JP, Steinhoff HJ, Goedecke A, Schrader J, Gladwin MT, Kelm M, Rassaf T. Nitrite reductase activity of myoglobin regulates respiration and cellular viability in myocardial ischemia-reperfusion injury. Proc Natl Acad Sci USA 105: 10256–10261, 2008. doi:10.1073/pnas.0801336105. 18632562 PMC2481313

[214] Rochon ER, Corti P. Globins and nitric oxide homeostasis in fish embryonic development. Mar Genomics 49: 100721, 2020. doi:10.1016/j.margen.2019.100721. 31711848

[215] Aboouf MA, Gorr TA, Hamdy NM, Gassmann M, Thiersch M. Myoglobin in brown adipose tissue: a multifaceted player in thermogenesis. Cells 12: 2240, 2023. doi:10.3390/cells12182240. 37759463 PMC10526770

[216] Bal NC, Singh S, Reis FC, Maurya SK, Pani S, Rowland LA, Periasamy M. Both brown adipose tissue and skeletal muscle thermogenesis processes are activated during mild to severe cold adaptation in mice. J Biol Chem 292: 16616–16625, 2017. doi:10.1074/jbc.M117.790451. 28794154 PMC5633124

[217] Fournier B, Murray B, Gutzwiller S, Marcaletti S, Marcellin D, Bergling S, Brachat S, Persohn E, Pierrel E, Bombard F, Hatakeyama S, Trendelenburg AU, Morvan F, Richardson B, Glass DJ, Lach-Trifilieff E, Feige JN. Blockade of the activin receptor IIB activates functional brown adipogenesis and thermogenesis by inducing mitochondrial oxidative metabolism. Mol Cell Biol 32: 2871–2879, 2012. doi:10.1128/MCB.06575-11. 22586266 PMC3416189

[218] Watanabe M, Yamamoto T, Kakuhata R, Okada N, Kajimoto K, Yamazaki N, Kataoka M, Baba Y, Tamaki T, Shinohara Y. Synchronized changes in transcript levels of genes activating cold exposure-induced thermogenesis in brown adipose tissue of experimental animals. Biochim Biophys Acta 1777: 104–112, 2008. doi:10.1016/j.bbabio.2007.10.014. 18036333

[219] Amieux PS, McKnight GS. Cyclic nucleotides converge on brown adipose tissue differentiation. Sci Signal 3: pe2, 2010. doi:10.1126/scisignal.3104pe2. 20068229

[220] Nisoli E, Clementi E, Tonello C, Sciorati C, Briscini L, Carruba MO. Effects of nitric oxide on proliferation and differentiation of rat brown adipocytes in primary cultures. Br J Pharmacol 125: 888–894, 1998. doi:10.1038/sj.bjp.0702131. 9831929 PMC1571007

[221] Mitschke MM, Hoffmann LS, Gnad T, Scholz D, Kruithoff K, Mayer P, Haas B, Sassmann A, Pfeifer A, Kilic A. Increased cGMP promotes healthy expansion and browning of white adipose tissue. FASEB J 27: 1621–1630, 2013. doi:10.1096/fj.12-221580. 23303211

[222] Haas B, Mayer P, Jennissen K, Scholz D, Berriel Diaz M, Bloch W, Herzig S, Fässler R, Pfeifer A. Protein kinase G controls brown fat cell differentiation and mitochondrial biogenesis. Sci Signal 2: ra78, 2009. doi:10.1126/scisignal.2000511. 19952371

[223] Balkow A, Jagow J, Haas B, Siegel F, Kilić A, Pfeifer A. A novel crosstalk between Alk7 and cGMP signaling differentially regulates brown adipocyte function. Mol Metab 4: 576–583, 2015. doi:10.1016/j.molmet.2015.06.003. 26266090 PMC4529496

[224] Szelényi Z. Effect of cold exposure on oxygen tension in brown adipose tissue in the non-cold adapted adult rat. Acta Physiol Acad Sci Hung 33: 311–316, 1968. 5679804

[225] Shaw CS, Clark J, Wagenmakers AJ. The effect of exercise and nutrition on intramuscular fat metabolism and insulin sensitivity. Annu Rev Nutr 30: 13–34, 2010. doi:10.1146/annurev.nutr.012809.104817. 20373917

[226] McCoin CS, Knotts TA, Adams SH. Acylcarnitines—old actors auditioning for new roles in metabolic physiology. Nat Rev Endocrinol 11: 617–625, 2015. doi:10.1038/nrendo.2015.129. 26303601 PMC4966159

[227] Brøns C, Grunnet LG. Mechanisms in endocrinology: skeletal muscle lipotoxicity in insulin resistance and type 2 diabetes: a causal mechanism or an innocent bystander? Eur J Endocrinol 176: R67–R78, 2017. doi:10.1530/EJE-16-0488. 27913612

[228] Spector AA. Fatty acid binding to plasma albumin. J Lipid Res 16: 165–179, 1975. doi:10.1016/S0022-2275(20)36723-7. 236351

[229] Smathers RL, Petersen DR. The human fatty acid-binding protein family: evolutionary divergences and functions. Hum Genomics 5: 170–191, 2011. doi:10.1186/1479-7364-5-3-170. 21504868 PMC3500171

[230] Kader JC. Lipid-transfer proteins: a puzzling family of plant proteins. Trends Plant Sci 2: 66–70, 1997. doi:10.1016/S1360-1385(97)82565-4.

[231] Hostetler HA, Petrescu AD, Kier AB, Schroeder F. Peroxisome proliferator-activated receptor alpha interacts with high affinity and is conformationally responsive to endogenous ligands. J Biol Chem 280: 18667–18682, 2005. doi:10.1074/jbc.M412062200. 15774422

[232] Kaikaus RM, Chan WK, Ortiz de Montellano PR, Bass NM. Mechanisms of regulation of liver fatty acid-binding protein. Mol Cell Biochem 123: 93–100, 1993. doi:10.1007/BF01076479. 8232272

[233] Colella M, Lobasso S, Babudri F, Corcelli A. Palmitic acid is associated with halorhodopsin as a free fatty acid. Biochim Biophys Acta 1370: 273–279, 1998. doi:10.1016/S0005-2736(97)00276-9. 9545581

[234] Shih L, Chung Y, Sriram R, Jue T. Palmitate interaction with physiological states of myoglobin. Biochim Biophys Acta 1840: 656–666, 2014. doi:10.1016/j.bbagen.2013.10.028. 24482816 PMC3934850

[235] Shih L, Chung Y, Sriram R, Jue T. Interaction of myoglobin with oleic acid. Chem Phys Lipids 191: 115–122, 2015. doi:10.1016/j.chemphyslip.2015.07.010. 26220615 PMC4633329

[236] Simard JR, Zunszain PA, Hamilton JA, Curry S. Location of high and low affinity fatty acid binding sites on human serum albumin revealed by NMR drug-competition analysis. J Mol Biol 361: 336–351, 2006. doi:10.1016/j.jmb.2006.06.028. 16844140

[237] Curry S, Mandelkow H, Brick P, Franks N. Crystal structure of human serum albumin complexed with fatty acid reveals an asymmetric distribution of binding sites. Nat Struct Biol 5: 827–835, 1998. doi:10.1038/1869. 9731778

[238] Bhattacharya AA, Grüne T, Curry S. Crystallographic analysis reveals common modes of binding of medium and long-chain fatty acids to human serum albumin. J Mol Biol 303: 721–732, 2000. doi:10.1006/jmbi.2000.4158. 11061971

[239] Zhu TT, Zhang Y, Luo XA, Wang SZ, Jia MQ, Chen ZX. Difference in binding of long- and medium-chain fatty acids with serum albumin: the role of macromolecular crowding effect. J Agric Food Chem 66: 1242–1250, 2018. doi:10.1021/acs.jafc.7b03548. 29303261

[240] Petitpas I, Grüne T, Bhattacharya AA, Curry S. Crystal structures of human serum albumin complexed with monounsaturated and polyunsaturated fatty acids. J Mol Biol 314: 955–960, 2001. doi:10.1006/jmbi.2000.5208. 11743713

[241] Glatz JF, Veerkamp JH. A radiochemical procedure for the assay of fatty acid binding by proteins. Anal Biochem 132: 89–95, 1983. doi:10.1016/0003-2697(83)90429-3. 6194713

[242] Zelencova-Gopejenko D, Videja M, Grandane A, Pudnika-Okinčica L, Sipola A, Vilks K, Dambrova M, Jaudzems K, Liepinsh E. Heart-type fatty acid binding protein binds long-chain acylcarnitines and protects against lipotoxicity. Int J Mol Sci 24: 5528, 2023. doi:10.3390/ijms24065528. 36982599 PMC10058761

[243] Schulenberg‐Schell H, Schäfer P, Keuper HJ, Stanislawski B, Hoffmann E, Rüterjans H, Spener F. Interactions of fatty acids with neutral fatty‐acid‐binding protein from bovine liver. Eur J Biochem 170: 565–574, 1988. doi:10.1111/j.1432-1033.1988.tb13735.x. 3338452

[244] Aguer C, McCoin CS, Knotts TA, Thrush AB, Ono-Moore K, McPherson R, Dent R, Hwang DH, Adams SH, Harper ME. Acylcarnitines: potential implications for skeletal muscle insulin resistance. FASEB J 29: 336–345, 2015. doi:10.1096/fj.14-255901. 25342132 PMC4285541

[245] Rutkowsky JM, Knotts TA, Ono-Moore KD, McCoin CS, Huang S, Schneider D, Singh S, Adams SH, Hwang DH. Acylcarnitines activate proinflammatory signaling pathways. Am J Physiol Endocrinol Metab 306: E1378–E1387, 2014. doi:10.1152/ajpendo.00656.2013. 24760988 PMC4059985

[246] McCoin CS, Knotts TA, Ono-Moore KD, Oort PJ, Adams SH. Long-chain acylcarnitines activate cell stress and myokine release in C2C12 myotubes: calcium-dependent and -independent effects. Am J Physiol Endocrinol Metab 308: E990–E1000, 2015. doi:10.1152/ajpendo.00602.2014. 25852008 PMC4451287

[247] Blackburn ML, Ono-Moore KD, Sobhi HF, Adams SH. Carnitine palmitoyltransferase 2 knockout potentiates palmitate-induced insulin resistance in C2C12 myotubes. Am J Physiol Endocrinol Metab 319: E265–E275, 2020. doi:10.1152/ajpendo.00515.2019. 32459525

[248] Liepinsh E, Kuka J, Vilks K, Svalbe B, Stelfa G, Vilskersts R, Sevostjanovs E, Goldins NR, Groma V, Grinberga S, Plaas M, Makrecka-Kuka M, Dambrova M. Low cardiac content of long-chain acylcarnitines in TMLHE knockout mice prevents ischaemia-reperfusion-induced mitochondrial and cardiac damage. Free Radic Biol Med 177: 370–380, 2021. doi:10.1016/j.freeradbiomed.2021.10.035. 34728372

[249] Armbruster J, Aboouf MA, Gassmann M, Egert A, Schorle H, Hornung V, Schmidt T, Schmid-Burgk JL, Kristiansen G, Bicker A, Hankeln T, Zhu H, Gorr TA. Myoglobin regulates fatty acid trafficking and lipid metabolism in mammary epithelial cells. PLoS One 17: e0275725, 2022. doi:10.1371/journal.pone.0275725. 36223378 PMC9555620

[250] Reis A, Spickett CM. Chemistry of phospholipid oxidation. Biochim Biophys Acta 1818: 2374–2387, 2012. doi:10.1016/j.bbamem.2012.02.002. 22342938

[251] Volinsky R, Kinnunen PK. Oxidized phosphatidylcholines in membrane-level cellular signaling: from biophysics to physiology and molecular pathology. FEBS J 280: 2806–2816, 2013. doi:10.1111/febs.12247. 23506295

[252] Kanner J, German JB, Kinsella JE. Initiation of lipid peroxidation in biological systems. Crit Rev Food Sci Nutr 25: 317–364, 1987. doi:10.1080/10408398709527457. 3304843

[253] Apte S, Morrissey PA. Effect of haemoglobin and ferritin on lipid oxidation in raw and cooked muscle systems. Food Chem 25: 127–134, 1987. doi:10.1016/0308-8146(87)90061-6.

[254] Baron CP, Skibsted LH, Andersen HJ. Prooxidative activity of myoglobin species in linoleic acid emulsions. J Agric Food Chem 45: 1704–1710, 1997. doi:10.1021/jf960639f.

[255] Richards MP, Hultin HO. Effect of pH on lipid oxidation using trout hemolysate as a catalyst: a possible role for deoxyhemoglobin. J Agric Food Chem 48: 3141–3147, 2000. doi:10.1021/jf991059w. 10956082

[256] Goodman DS. The interaction of human serum albumin with long-chain fatty acid anions. J Am Chem Soc 80: 3892–3898, 1958. doi:10.1021/ja01548a024.

[257] Decker EA, Hultin HO. Lipid oxidation in muscle foods via redox iron. In: *Lipid Oxidation in Food*. Washington, DC: ACS Publications, 1992, p. 33–54.

[258] Kanner J. Oxidative processes in meat and meat products: quality implications. Meat Sci 36: 169–189, 1994. doi:10.1016/0309-1740(94)90040-X. 22061459

[259] Kaschnitz RM, Hatefi Y. Lipid oxidation in biological membranes. Arch Biochem Biophys 171: 292–304, 1975. doi:10.1016/0003-9861(75)90036-3. 242270

[260] Baron CP, Skibsted LH, Andersen HJ. Peroxidation of linoleate at physiological pH: hemichrome formation by substrate binding protects against metmyoglobin activation by hydrogen peroxide. Free Radic Biol Med 28: 549–558, 2000. doi:10.1016/S0891-5849(99)00240-3. 10719236

[261] Rodríguez-Malaver AJ, Leake DS, Rice-Evans CA. The effects of pH on the oxidation of low‐density lipoprotein by copper and metmyoglobin are different. FEBS Lett 406: 37–41, 1997. doi:10.1016/S0014-5793(97)00233-0. 9109382

[262] Stewart JM. Free fatty acids enhance the oxidation of oxymyoglobin and inhibit the peroxidase activity of metmyoglobin. Biochem Cell Biol 68: 1096–1102, 1990. doi:10.1139/o90-164. 2175198

[263] Hirano Y, Olcott HS. Effect of heme compounds on lipid oxidation. J Am Oil Chem Soc 48: 523–524, 1971. doi:10.1007/BF02544553. 5111840

[264] Hogg N, Rice-Evans C, Darley-Usmar V, Wilson MT, Paganga G, Bourne L. The role of lipid hydroperoxides in the myoglobin-dependent oxidation of LDL. Arch Biochem Biophys 314: 39–44, 1994. doi:10.1006/abbi.1994.1409. 7944405

[265] Kanner J, Harel S. Lipid peroxidation and oxidation of several compounds by H_2_O_2_ activated metmyoglobin. Lipids 20: 625–628, 1985. doi:10.1007/BF02534290. 4046748

[266] Kanner J, Harel S. Initiation of membranal lipid peroxidation by activated metmyoglobin and methemoglobin. Arch Biochem Biophys 237: 314–321, 1985. doi:10.1016/0003-9861(85)90282-6. 3977316

[267] Rao SI, Wilks A, Hamberg M, Ortiz de Montellano PR. The lipoxygenase activity of myoglobin. Oxidation of linoleic acid by the ferryl oxygen rather than protein radical. J Biol Chem 269: 7210–7216, 1994. doi:10.1016/S0021-9258(17)37269-1. 8125933

[268] Akhrem AA, Andreyuk GM, Kisel MA, Kiselev PA. Hemoglobin conversion to hemichrome under the influence of fatty acids. Biochim Biophys Acta 992: 191–194, 1989. doi:10.1016/0304-4165(89)90009-3. 2503042

[269] Bourne LC, Lamb DJ, Collis CS, O’Brien M, Leake DS, Rice-Evans C. Non‐oxidative modification of low density lipoprotein by ruptured myocytes. FEBS Lett 414: 576–580, 1997. doi:10.1016/S0014-5793(97)01075-2. 9323039

[270] Lee TY. Lactate: a multifunctional signaling molecule. Yeungnam Univ J Med 38: 183–193, 2021. doi:10.12701/yujm.2020.00892. 33596629 PMC8225492

[271] Voet D, Voet GJ, Pratt WC. Fundamentals of Biochemistry: Life at the Molecular Level (5th ed.). Hoboken, NY: Wiley, 2016.

[272] Passarella S, de Bari L, Valenti D, Pizzuto R, Paventi G, Atlante A. Mitochondria and l-lactate metabolism. FEBS Lett 582: 3569–3576, 2008. doi:10.1016/j.febslet.2008.09.042. 18831974

[273] Pagliarini DJ, Calvo SE, Chang B, Sheth SA, Vafai SB, Ong SE, Walford GA, Sugiana C, Boneh A, Chen WK, Hill DE, Vidal M, Evans JG, Thorburn DR, Carr SA, Mootha VK. A mitochondrial protein compendium elucidates complex I disease biology. Cell 134: 112–123, 2008. doi:10.1016/j.cell.2008.06.016. 18614015 PMC2778844

[274] Hashimoto T, Masuda S, Taguchi S, Brooks GA. Immunohistochemical analysis of MCT1, MCT2 and MCT4 expression in rat plantaris muscle. J Physiol 567: 121–129, 2005. doi:10.1113/jphysiol.2005.087411. 15932892 PMC1474173

[275] Hashimoto T, Hussien R, Cho HS, Kaufer D, Brooks GA. Evidence for the mitochondrial lactate oxidation complex in rat neurons: demonstration of an essential component of brain lactate shuttles. PLoS One 3: e2915, 2008. doi:10.1371/journal.pone.0002915. 18698340 PMC2488371

[276] Hashimoto T, Hussien R, Brooks GA. Colocalization of MCT1, CD147, and LDH in mitochondrial inner membrane of L6 muscle cells: evidence of a mitochondrial lactate oxidation complex. Am J Physiol Endocrinol Metab 290: E1237–E1244, 2006. doi:10.1152/ajpendo.00594.2005. 16434551

[277] Dubouchaud H, Butterfield GE, Wolfel EE, Bergman BC, Brooks GA. Endurance training, expression, and physiology of LDH, MCT1, and MCT4 in human skeletal muscle. Am J Physiol Endocrinol Metab 278: E571–E579, 2000. doi:10.1152/ajpendo.2000.278.4.E571. 10751188

[278] Brooks GA, Dubouchaud H, Brown M, Sicurello JP, Butz CE. Role of mitochondrial lactate dehydrogenase and lactate oxidation in the intracellular lactate shuttle. Proc Natl Acad Sci USA 96: 1129–1134, 1999. doi:10.1073/pnas.96.3.1129. 9927705 PMC15362

[279] Nelson DL, Cox MM. Lenhninger Principles of Biochemistry (7th ed.). New York: W. H. Freeman, 2017.

[280] Brooks GA. Lactate as a fulcrum of metabolism. Redox Biol 35: 101454, 2020. doi:10.1016/j.redox.2020.101454. 32113910 PMC7284908

[281] Henderson GC, Horning MA, Wallis GA, Brooks GA. Pyruvate metabolism in working human skeletal muscle. Am J Physiol Endocrinol Metab 292: E366, 2007. doi:10.1152/ajpendo.00363.2006. 16926378

[282] Proia P, Di Liegro CM, Schiera G, Fricano A, Di Liegro I. Lactate as a metabolite and a regulator in the central nervous system. Int J Mol Sci 17: 1450, 2016. doi:10.3390/ijms17091450. 27598136 PMC5037729

[283] Rogatzki MJ, Ferguson BS, Goodwin ML, Gladden LB. Lactate is always the end product of glycolysis. Front Neurosci 9: 22, 2015. doi:10.3389/fnins.2015.00022. 25774123 PMC4343186

[284] Khacho M, Tarabay M, Patten D, Khacho P, MacLaurin JG, Guadagno J, Bergeron R, Cregan SP, Harper ME, Park DS, Slack RS. Acidosis overrides oxygen deprivation to maintain mitochondrial function and cell survival. Nat Commun 5: 3550, 2014. doi:10.1038/ncomms4550. 24686499 PMC3988820

[285] Ascenzi P, Bocedi A, Bolli A, Fasano M, Notari S, Polticelli F. Allosteric modulation of monomeric proteins. Biochem Mol Biol Educ 33: 169–176, 2005. doi:10.1002/bmb.2005.494033032470. 21638571

[286] Adepu KK, Bhandari D, Anishkin A, Adams SH, Chintapalli SV. Myoglobin interaction with lactate rapidly releases oxygen: studies on binding thermodynamics, spectroscopy, and oxygen kinetics. Int J Mol Sci 23: 4747, 2022. doi:10.3390/ijms23094747. 35563138 PMC9103699

[287] Arandjelovic O. On the value of c: can low affinity systems be studied by isothermal titration calorimetry. Int J Appl Philos 35: 227–241, 2021. doi:10.5840/ijap2022422169.

[288] Fukui H, Taniguchi S, Ueta Y, Yoshida A, Ohtahara A, Hisatome I, Shigemasa C. Enhanced activity of the purine nucleotide cycle of the exercising muscle in patients with hyperthyroidism. J Clin Endocrinol Metab 86: 2205–2210, 2001. doi:10.1210/jcem.86.5.7516. 11344228

[289] Constantin-Teodosiu D, Peirce NS, Fox J, Greenhaff PL. Muscle pyruvate availability can limit the flux, but not activation, of the pyruvate dehydrogenase complex during submaximal exercise in humans. J Physiol 561: 647–655, 2004. doi:10.1113/jphysiol.2004.073411. 15579544 PMC1665350

[290] Ong HY, O’Dochartaigh CS, Lovell S, Patterson VH, Wasserman K, Nicholls DP, Riley MS. Gas exchange responses to constant work-rate exercise in patients with glycogenosis type V and VII. Am J Respir Crit Care Med 169: 1238–1244, 2004. doi:10.1164/rccm.200307-974OC. 15070817

[291] Valentovic MA, Minigh J. Pyruvate attenuates myoglobin in vitro toxicity. Toxicol Sci 74: 345–351, 2003. doi:10.1093/toxsci/kfg135. 12773762

[292] Fraser J, de Mello LV, Ward D, Rees HH, Williams DR, Fang Y, Brass A, Gracey AY, Cossins AR. Hypoxia-inducible myoglobin expression in nonmuscle tissues. Proc Natl Acad Sci USA 103: 2977–2981, 2006. doi:10.1073/pnas.0508270103. 16469844 PMC1413783

[293] Kanatous SB, Mammen PP. Regulation of myoglobin expression. J Exp Biol 213: 2741–2747, 2010. doi:10.1242/jeb.041442. 20675543 PMC2912755

[294] Kanatous SB, DiMichele LV, Cowan DF, Davis RW. High aerobic capacities in the skeletal muscles of pinnipeds: adaptations to diving hypoxia. J Appl Physiol (1985) 86: 1247–1256, 1999. doi:10.1152/jappl.1999.86.4.1247. 10194210

[295] Kanatous SB, Mammen PP, Rosenberg PB, Martin CM, White MD, Dimaio JM, Huang G, Muallem S, Garry DJ. Hypoxia reprograms calcium signaling and regulates myoglobin expression. Am J Physiol Cell Physiol 296: C393–C402, 2009. doi:10.1152/ajpcell.00428.2008. 19005161 PMC2660263

[296] Kleinert M, Parker BL, Jensen TE, Raun SH, Pham P, Han X, James DE, Richter EA, Sylow L. Quantitative proteomic characterization of cellular pathways associated with altered insulin sensitivity in skeletal muscle following high-fat diet feeding and exercise training. Sci Rep 8: 10723, 2018. doi:10.1038/s41598-018-28540-5. 30013070 PMC6048112

[297] Mizunoya W, Iwamoto Y, Shirouchi B, Sato M, Komiya Y, Razin FR, Tatsumi R, Sato Y, Nakamura M, Ikeuchi Y. Dietary fat influences the expression of contractile and metabolic genes in rat skeletal muscle. PLoS One 8: e80152, 2013. doi:10.1371/journal.pone.0080152. 24244634 PMC3823866

[298] Mizunoya W, Sawano S, Iwamoto Y, Sato Y, Tatsumi R, Ikeuchi Y. Effect of 48-h food deprivation on the expressions of myosin heavy-chain isoforms and fiber type-related factors in rats. J Nutr Sci Vitaminol (Tokyo) 59: 289–298, 2013. doi:10.3177/jnsv.59.289. 24064729

[299] Helfenrath K, Sauer M, Kamga M, Wisniewsky M, Burmester T, Fabrizius A. The more, the merrier? Multiple myoglobin genes in fish species, especially in gray bichir (*Polypterus senegalus*) and reedfish (*Erpetoichthys calabaricus*). Genome Biol Evol 13: evab078, 2021. doi:10.1093/gbe/evab078. 33871590 PMC8480196

[300] Bicker A, Brahmer AM, Meller S, Kristiansen G, Gorr TA, Hankeln T. The distinct gene regulatory network of myoglobin in prostate and breast cancer. PLoS One 10: e0142662, 2015. doi:10.1371/journal.pone.0142662. 26559958 PMC4641586

[301] Bicker A, Dietrich D, Gleixner E, Kristiansen G, Gorr TA, Hankeln T. Extensive transcriptional complexity during hypoxia-regulated expression of the myoglobin gene in cancer. Hum Mol Genet 23: 479–490, 2014. doi:10.1093/hmg/ddt438. 24026678

[302] Meller S, Bicker A, Montani M, Ikenberg K, Rostamzadeh B, Sailer V, Wild P, Dietrich D, Uhl B, Sulser T, Moch H, Gorr TA, Stephan C, Jung K, Hankeln T, Kristiansen G. Myoglobin expression in prostate cancer is correlated to androgen receptor expression and markers of tumor hypoxia. Virchows Arch 465: 419–427, 2014. doi:10.1007/s00428-014-1646-y. 25172328

[303] El-Tohamy R, Elkholi I, Elsherbiny ME, Magdy M, Hammam O, Allalunis-Turner J, Emara M. Myoglobin variants are expressed in human glioblastoma cells—hypoxia effect? Oncol Rep 43: 975–985, 2020. doi:10.3892/or.2020.7479. 32020230

[304] Elsherbiny ME, Shaaban M, El-Tohamy R, Elkholi IE, Hammam OA, Magdy M, Allalunis-Turner J, Emara M. Expression of myoglobin in normal and cancer brain tissues: correlation with hypoxia markers. Front Oncol 11: 590771, 2021. doi:10.3389/fonc.2021.590771. 33996536 PMC8120281

[305] Meller S, Van Ellen A, Gevensleben H, Bicker A, Hankeln T, Gorr TA, Sailer V, Dröge F, Schröck F, Bootz F, Schröck A, Perner S, Dietrich D, Kristiansen G. Ectopic myoglobin expression is associated with a favourable outcome in head and neck squamous cell carcinoma patients. Anticancer Res 36: 6235–6241, 2016. doi:10.21873/anticanres.11217. 27919941

[306] Scrima R, Agriesti F, Pacelli C, Piccoli C, Pucci P, Amoresano A, Cela O, Nappi L, Tataranni T, Mori G, Formisano P, Capitanio N. Myoglobin expression by alternative transcript in different mesenchymal stem cells compartments. Stem Cell Res Ther 13: 209, 2022. doi:10.1186/s13287-022-02880-6. 35598009 PMC9123686

